# Dendritic morphology, synaptic transmission, and activity of mature granule cells born following pilocarpine-induced status epilepticus in the rat

**DOI:** 10.3389/fncel.2015.00384

**Published:** 2015-10-07

**Authors:** Fei Gao, Xueying Song, Dexiao Zhu, Xiaochen Wang, Aijun Hao, J. Victor Nadler, Ren-Zhi Zhan

**Affiliations:** ^1^Department of Physiology, Shandong University School of MedicineJinan, China; ^2^Department of Histology and Embryology, Shandong University School of MedicineJinan, China; ^3^Departments of Pharmacology and Neurobiology, Duke University Medical CenterDurham, NC, USA

**Keywords:** activity-regulated cytoskeleton-associated protein, dendritic spine, epilepsy, neurogenesis, retroviral vector, Sholl analysis

## Abstract

To understand the potential role of enhanced hippocampal neurogenesis after pilocarpine-induced status epilepticus (SE) in the development of epilepsy, we quantitatively analyzed the geometry of apical dendrites, synaptic transmission, and activation levels of normotopically distributed mature newborn granule cells in the rat. SE in male Sprague-Dawley rats (between 6 and 7 weeks old) lasting for more than 2 h was induced by an intraperitoneal injection of pilocarpine. The complexity, spine density, miniature post-synaptic currents, and activity-regulated cytoskeleton-associated protein (Arc) expression of granule cells born 5 days after SE were studied between 10 and 17 weeks after CAG-GFP retroviral vector-mediated labeling. Mature granule cells born after SE had dendritic complexity similar to that of granule cells born naturally, but with denser mushroom-like spines in dendritic segments located in the outer molecular layer. Miniature inhibitory post-synaptic currents (mIPSCs) were similar between the controls and rats subjected to SE; however, smaller miniature excitatory post-synaptic current (mEPSC) amplitude with a trend toward less frequent was found in mature granule cells born after SE. After maturation, granule cells born after SE did not show denser Arc expression in the resting condition or 2 h after being activated by pentylenetetrazol-induced transient seizure activity than vicinal GFP-unlabeled granule cells. Thus our results suggest that normotopic granule cells born after pilocarpine-induced SE are no more active when mature than age-matched, naturally born granule cells.

## Introduction

Within days after seizure activity, especially after status epilepticus (SE), neurogenesis is enhanced in the hippocampal dentate gyrus (Parent et al., 1997; Gray and Sundstrom, 1998; Scott et al., 1998; Scharfman et al., 2000). Newborn granule cells survive (Bengzon et al., 1997; Ekdahl et al., 2001, 2003) and mature to become long-term residents in the dentate gyrus (Parent et al., 1997; Scharfman et al., 2000). According to the locations to which newborn granule cells migrate, granule cells born after SE can be divided into normotopic and ectopic populations. Most ectopically located granule cells are in the hilus (Parent et al., 1997; Scharfman et al., 2000; Dashtipour et al., 2001), but a small portion of them are present in the molecular layer (Liang et al., 2013). Analyzing hilar ectopic granule cells has revealed that not all, but a certain portion of them differ morphologically and electrophysiologically from normotopic granule cells (Scharfman et al., 2000; Zhan and Nadler, 2009; Zhan et al., 2010; Cameron et al., 2011; Althaus et al., 2015) in ways that suggest ectopic granule cells born after SE contribute to the development of epilepsy.

Newborn granule cells that migrate to the granule cell layer after SE are far more numerous than ectopic granule cells. Morphological, electrophysiological, and functional studies aimed at exploring the influence of normotopic newborn granule cells in the development of epilepsy have reached somewhat conflicting conclusions (Bielefeld et al., 2014). Although morphological studies done in different rodent epilepsy models have found that normotopic granule cells born after SE are morphologically different from naturally born granule cells in several respects (Kron et al., 2010; Murphy et al., 2011, 2012; Hester and Danzer, 2013), electrophysiological studies have not proven that normotopic newborn granule cells are more excitable than naturally born, age-matched granule cells (Jakubs et al., 2006; Wood et al., 2011). As the adult hippocampal neurogenesis plays an important role in learning, memory formation, and mood regulation (Deng et al., 2010; Samuels and Hen, 2011), illustrating the structural and functional differences between granule cells born after SE and those born naturally would also be helpful in understanding the role of enhanced neurogenesis not only for the development of epilepsy but also for the occurrence of comorbid cognitive impairment and behavioral disturbances (Chauvière et al., 2009; Cavarsan et al., 2013).

By applying retroviral vector-mediated cell labeling, we compared mature granule cells born 5 days after SE to those naturally born with respect to dendritic morphology, intrinsic membrane properties, synaptic connectivity, and magnitude of cellular activity in the resting condition and after being activated by transient seizure activity. The magnitude of cellular activity was indicated by the expression of activity-regulated cytoskeletal-associated protein (Arc), which has been widely used as a marker of neuronal activation (Guzowski et al., 2005; Shepherd and Bear, 2011).

## Materials and methods

### Animals

All animal procedures were approved by the Animal Ethics Committee of Shandong University School of Medicine and performed in accordance with the guidelines set by US National Research Council (2011). Male Sprague-Dawley rats weighing between 150 and 175 g (between 6 and 7 weeks old) were purchased from the Animal Center of Shandong University of Traditional Chinese Medicine (Jinan, Shandong, China). Animals were housed in standard rodent cages under a constant 12 h light/dark cycle with free access to food and water for at least 7 days before experiments.

### Construction of the CAG-GFP retroviral vector

Two packing cell lines coupled with plasmids pCAG-GFP and pCMV-VSV-G (Addgene, Cambridge, MA, USA) were used to construct the CAG-GFP retroviral vector. First, Platinum-GP cells (a gift from Dr. Guo at Ocean University of China) were co-transfected by pCAG-GFP and pCMV-VSV-G with the facilitation of lipofectamine 2000 (Invitrogen, Carlsbad, CA, USA). Supernatants collected at 48 and 72 h after transfection were pooled together and filtered through a 0.45 μm syringe filter. The filtered supernatant was centrifuged at 65,000 g for 2 h by using an Optima L-100XP Beckman ultraspeed centrifuge with the rotor SW70 Ti (Beckman Coulter, Brea, CA, USA) at 4°C. The virus-containing pellet was then suspended in 0.1 M phosphate-buffered saline (PBS), transferred into a new tube that was centrifuged for another 2 h at 4°C with the rotor SW41 Ti (Beckman Coulter, Brea, CA, USA). The resulted pellet was re-suspended in 0.1 M PBS and used to infect platinum-E cells (a gift from Dr. Dong of Nanjing Agricultural University) for generating a stable virus-producing cell line. The virus-producing platinum-E cells were frozen at −80°C. Concentrated virus solution was then obtained from the stable virus-producing cells through two-step ultraspeed centrifugation as mentioned above.

### Pilocarpine-induced status epilepticus

Status epilepticus was induced by intraperitoneal injection of pilocarpine as described previously (Zhan and Nadler, 2009). Rats were pretreated with an intraperitoneal injection of mixed scopolamine methyl bromide (2 mg/kg) and terbutaline hemisulfate (2 mg/kg) which were purchased from Sigma (St. Louis, MO, USA). Thirty minutes later, 340 mg/kg of pilocarpine hydrochloride (MP Biomedicals, Solon, OH, USA) was intraperitoneally injected. Animals were chosen for further studies if the continuous seizure activity was at Racine stage two or above (Racine, 1972) and lasted for at least 2 h. If seizure activity was not initiated until 30 min after the initial pilocarpine dose, an additional dose of 100 mg/kg was given. Control animals received an equal volume of normal saline instead of pilocarpine after the pretreatment with scopolamine methyl bromide and terbutaline hemisulfate.

### Intradentate injection of CAG-GFP retroviral vector

Intradentate injection of CAG-GFP retroviral vector was performed on the 5th day after SE induction or saline injection. After animals were anesthetized with an intraperitoneal injection of sodium pentobarbital (25 mg/kg) and ketamine (60 mg/kg), they were individually placed on a stereotaxic frame suitable for rats (Stoelting, Wood Dale, IL, USA). After the skin was sterilized with sequential applications of 0.5% povidone iodine and 75% alcohol, the scalp was incised and a skull hole on the right side was drilled. One microliter of CAG-GFP retroviral vector was injected into the right dentate gyrus at a rate of 0.1 μl /min with a 1 μl Hamilton syringe (Hamilton, Bonaduz, GR, Switzerland) connected to an automatic injection pump (Stoelting, Wood Dale, IL, USA). The injection site was located 3.2 mm posterior from bregma and 2.5 mm right of the midline at a depth of 2.4 mm from the surface of the brain. The needle was left in place for an additional 15 min after the completion of injection to prevent possible backflow before removal. All surgical procedures were performed under sterile conditions. Animals were returned to the cage after recovery from anesthesia. Examinations of newborn granule cells labeled by GFP were carried out at least 10 weeks (13.8 ± 0.3 weeks, range: 10–17 weeks) after vector injections.

### Transcardial perfusion and tissue preparation

At least 10 weeks after retroviral vector injection, 8 control rats and 8 SE rats were anesthetized with intraperitoneal injection of sodium pentobarbital (80 mg/kg). After complete paralysis, transcardial perfusion was done with heparinized normal saline (40–50 ml) followed by 2% paraformaldehyde in 0.1 M PBS. The brain was removed from the skull upon completion of transcardial perfusion. A tissue block made by trimming off the cerebellum and the frontal poles was post-fixed in the same fixative at 4°C overnight. Thereafter, the tissue block was sequentially immersed in 10% sucrose in 0.1 M PBS for 4 h, 15% sucrose in 0.1 M PBS for 8 h, and finally 20% sucrose in 0.1 M PBS overnight at 4°C. After the tissue block was firmly embedded with a medium that consisted of 30% (w/v) chicken egg albumin (Sigma, St. Louis, MO, USA), 0.5% (w/v) gelatin and 0.9% (v/v) glutaraldehyde in 0.1 M PBS as described previously (Zhan and Nadler, 2009), it was coronally cut through the hippocampi into 80 μm-thick-sections with a vibratome (VT1000S, Leica Biosystems, Nussloch, Germany). Sections in a range of approximately 1.5 mm posterior and anterior to the site of retroviral vector injection were collected and immunofluorescently stained with a rabbit anti-GFP as described below.

### Enhancement of GFP fluorescence with ANTI-GFP antibody

Free-floating sections were first washed with 0.1 M PBS, and then incubated in a blocking solution that consisted of 2.5% BSA, 0.2% Triton X-100 and 5% donkey serum in 0.1 M PBS at 4°C for 2.5 h to minimize the background stain. Thereafter, sections were incubated at 4°C overnight with a rabbit anti-GFP (Invitrogen, Carlsbad, CA, USA) that was diluted with the blocking solution. After the sections were rinsed with 0.1 M PBS (15 min × 3 times), the sections were incubated with an Alexa fluor 488-conjugated donkey anti-rabbit antibody (Invitrogen, Carlsbad, CA, USA) in a dilution of 1:600. After washing, sections were mounted on glass slides with 75% glycerol in PBS and coverslipped.

### Dendritic arborization analyses of newborn granule cells

The complexity of dendritic trees in newborn granule cells labeled by GFP was quantitated by Sholl analysis. Sections of 80 μm in thickness were prepared and the GFP signals were immunofluorescently amplified to prevent rapid bleaching as described above. Z-series stack of 2 μm thick was taken in GFP-positive cells locating in the suprapyramidal blade with a 20X objective (zoom = 1). For each rat, 3 to 4 cells were scanned. Only neurons displaying intact dendritic arborization were analyzed. After GFP-positive soma and apical dendrites were traced under ImageJ (http://imagej.nih.gov/ij/) with the NeuronJ plugin (http://www.imagescience.org/meijering/software/neuronj//), Sholl analysis was carried out by using ImageJ with the “Sholl analysis” plugin. The interval between concentric circles was 25 μm with the center point at the soma.

### Quantification of dendritic spines in newborn granule cells

The immunofluorescently amplified sections were also used for quantitative analysis of dendritic spines. For each rat, three newborn granule cells located in the suprapyramidal blade were chosen for this analysis. Z-series stacks were made in six dendritic segments (approximately 50 μm each) each neuron with a 63x objective; three were in the middle molecular layer and another three were in the outer molecular layer. Other parameters set for z-stacks were as follows: zoom 4, 0.12 μm of z-series thickness, and a resolution of 0.03 μm × 0.03 μm × 0.12 μm. A 3-dimension image was reconstructed from individual z-stack files. Spines in each dendritic segment were marked and the numbers of mushroom-like spines and the rest were counted by using ImageJ. The densities of total and mushroom-like spines were calculated by dividing the numbers of spines with the length of dendritic segment. A spine was defined as a mushroom spine if the diameter of the head was greater than the width of the neck (Harris et al., 1992).

### Patch clamp analysis of GFP-labeled newborn granule cells

#### Acute hippocampal slice preparation

Hippocampal slices were prepared at least 10 weeks after intradentate injection of retroviral vector. Animals were individually anesthetized with ether and then decapitated. After removal, the brain was immersed in artificial cerebrospinal fluid (ACSF) that consisted of (in mM) 112 NaCl, 3.1 KCl, 1.8 CaCl2, 11.2 MgSO4, 0.4 KH2PO4, 26 NaHCO3, and 20 D-glucose equilibrated with 95% O2/5% CO2 (pH 7.35–7.45) at 6°C. The brain was then cut through the midline. The brain block containing the right hippocampus was cut into 410 μm-thick coronal slices with a VT 1200S vibratome (Leica Biosystems, Nussloch, Germany). The sections were then incubated in a 95% O2/5% CO2-saturated ACSF as mentioned above at 34.5°C for 30 min. Thereafter, sections were stored in the standard ACSF that consisted of (in mM) 122 NaCl, 3.1 KCl, 1.8 CaCl2, 1.2 MgSO4, 0.4 KH2PO4, 26 NaHCO3, and 20 D-glucose, equilibrated with 95% O2/5% CO2 at room temperature.

#### Slice perfusion, cell visualization, and patch clamp recording

Each slice was transferred to a RC-26 recording chamber (Warner Instruments, Hamden, CT, USA) and continuously perfused with 95% O2/5% CO2-equilibrated standard ACSF (2.5 ml/min) at room temperature. Slices were visualized with an Olympus BX51WI fluorescence microscope (Olympus, Tokyo, Japan), equipped with the far infrared-differential interference contrast (DIC) optics, a charge-coupled device camera (Qimaging, Surrey, BC, Canada), and a 40X water-immersion objective. After a GFP-positive granule cell was fluorescently identified, the cell was switched to DIC visualization and patch-recorded. Patch electrodes were pulled from borosilicate glass (OD 1.5 mm, ID 1.1 mm) using a P97 or P1000 pipette puller (Sutter Instruments, Novato, CA, USA). The tip resistance was 6–9 MΩ. Electrical signals were recorded with an Axopatch 700B amplifier equipped with a 1440A Digidata (Molecular Devices, Sunnyvale, CA, USA). Data were filtered at 2 kHz, digitized at 5 kHz, and stored using pCLAMP software (Molecular Devices, Sunnyvale, CA). Parameters recorded were spontaneous and evoked firing and miniature post-synaptic currents.

#### Spontaneous and evoked firing

To record spontaneous and evoked firing, we used a potassium gluconate-based internal solution, which contained (in mM): 115 potassium gluconate, 17 KCl, 10 HEPES, 0.1 EGTA, 2 ATP magnesium, 0.3 GTP Tris, 5 creatine phosphate, 20 U/ml creatine phosphokinase, and 0.8% of biocytin (all were from Sigma, St. Louis, MO, USA) (pH 7.25–7.30, adjusted with KOH; osmolarity 294–297 mOsm). After a high resistance seal (>2 GΩ) was formed, spontaneous firing was recorded for 5 min in cell-attached mode by holding the cell at −70 mV. Whole cell recording was then achieved through membrane rupture and the resting membrane potential was recorded. Membrane time constant, input resistance, and membrane capacitance were obtained from the current deflection in response to a 10 mV step hyperpolarization from the resting membrane potential for 500 ms. At least 5 min after membrane rupture, cellular firing pattern was determined by stepped current injection. The threshold potential, amplitude, width, amplitude of the after depolarization (ADP) and the ADP area of action potentials were calculated by analyzing the first action potential evoked by current injection. A burst firing was defined as the spikes appeared in trains containing not one but two or more spikes. A liquid junction potential of 10 mV was corrected.

#### Recording miniature inhibitory and excitatory post-synaptic currents

To record miniature synaptic currents, GFP-positive cells were patched and currents were recorded at −70 mV in the whole-cell mode at room temperature. The recording electrode was filled with an internal solution that contained (in mM): 126 CsCl, 10 HEPES, 5 EGTA, 2 MgATP, 1 MgCl2, 0.1 CaCl2, 5 creatine phosphate, 20 units/ml creatine phosphokinase, and 0.8% (wt/vol) biocytin (pH 7.25–7.30; osmolarity 293–297 mOsm). Miniature inhibitory postsynaptic current (mIPSC) were recorded in the presence of 1 μM tetrodotoxin (TTX), 10 μM 6-cyano-7-nitroquinoxaline-2,3-dione (CNQX), and 50 μM D-2-amino-5-phosphonopentanoate (D-AP5). Miniature excitatory postynaptic currents (mEPSCs) were recorded in the presence of 1 μM tetrodotoxin (TTX) and 30 μM bicuculline methiodide (Sigma, St. Louis, MO, USA). D-AP5, CNQX, and TTX were purchased from Tocris Bioscience (Bristol, UK). In each animal, only one cell was recorded. Recordings were made for 2.5 min and miniature post-synaptic current events were manually analyzed by using MiniAnalysis (Synaptosoft, For Lee, NJ, USA), as described previously (Zhan et al., 2010). Between the control and SE rats, the amplitude, frequency, 10–90% rise time, decay time, and area of current events were statistically compared.

### Activity of newborn granule cells following pilocarpine-induced status epilepticus

Because enhanced neuronal activity is associated with an increased Arc expression, its immunoreactivity was used to indicate the activation of newborn granule cells after pilocarpine-induced status epilepticus in the resting condition and in response to a transient seizure episode. At the 5th day after pilocarpine (SE) or saline injection (control), 1 μl of CAG-GFP retroviral vector was injected into the dentate gyrus to label SE-induced or naturally born granule cells with a protocol as mentioned above. At least 70 days after viral vector injection, pentylenetetrazol (PTZ) (Sigma, St. Louis, MO, USA) at a dose of 20 mg/kg was intraperitoneally injected to SE (PTZ-treated SEs) or control (PTZ-treated controls) rats. PTZ-untreated SE and PTZ-untreated control rats received an equal volume of saline. Diazepam at a dose of 5 mg/kg was intraperitoneally injected into rats 15 min after PTZ treatment or saline injection. Two hours after the administration of diazepam, rats were transcardially perfused with 4% paraformaldehyde in 0.1 M PBS. Arc was immunofluorescently stained in coronally cut sections (40 μm in thickness) in which at least one GFP-expressing soma was included. A rabbit anti-Arc purchased from Synaptic System (Goettingen, Germany) was diluted 1000 times. Alexa Flour 568-conjugated donkey anti-rabbit IgG (Invitrogen, Carlsbad, CA, USA) was used as a secondary antibody in a dilution of 1:600. Sections were scanned by using a Zeiss LSM 780 confocal microscope equipped with a 40 × objective at a zoom 1. The relative intensities of Arc immunoreactivity were measured in the GFP-positive soma and surrounding 10 GFP-negative granule cell-like somata by using ImageJ. The intensity of Arc immunoreactivity in the GFP-positive soma was normalized to the averaged Arc immunoreactivity in 10 surrounding GFP-negative granule cells.

### Statistical analysis

Quantitative data are expressed as mean ± standard error of the mean. In morphological data, the numbers of sampling are the numbers of animals studied, but the numbers of sampling in the electrophysiological studies are the numbers of cells recorded. Data yielded from two groups are compared with unpaired *t*-test except for the percentage of cells displaying spontaneous firing that was statistically compared with Chi-square test. Multiple group data are analyzed by One-Way variance analysis (ANOVA) followed by Newman–Keuls post-hoc test when it is necessary. The data of Sholl analysis are analyzed with repeated measure ANOVA. A *P* ≤ 0.05 is considered to be statistically different.

## Results

### Features of cell labeling with CAG-GFP retroviral vector in the rat hippocampal dentate gyrus

**Figure 1:**
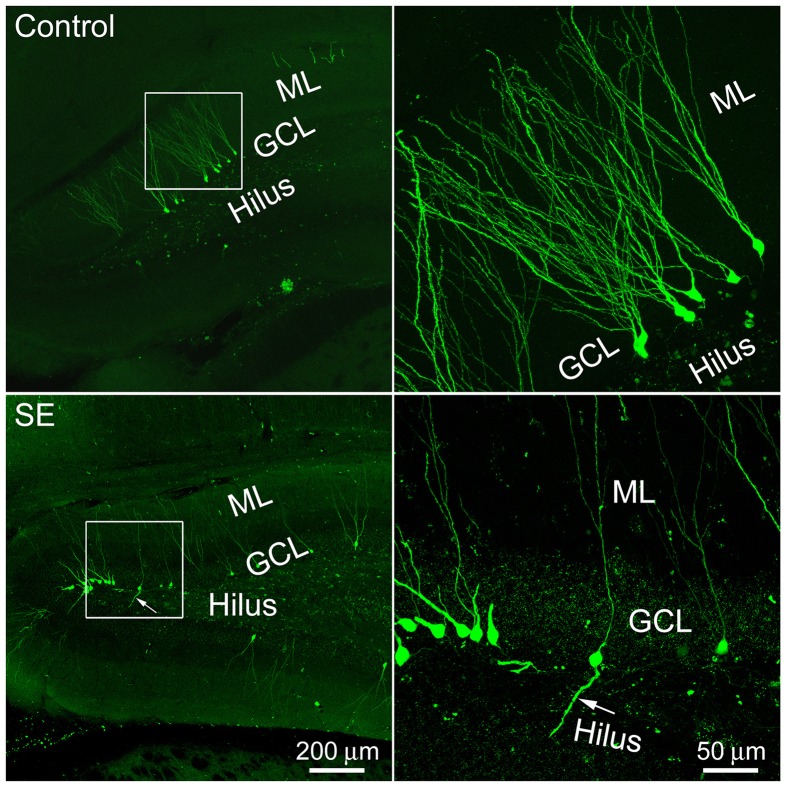
**Representative images show 4-month old newborn granule cells labeled by the CAG-GFP retroviral vector in the control and SE rats**. Rats were sacrificed 4 months after the CAG-GFP retroviral vector injection and coronal sections through the right hippocampus were cut into 80 μm. Z-series stacks of 2 μm were taken by a Zeiss LSM 780 confocal microscope. The white box-indicated regions in the left panels were scanned under a higher objective and are shown in the right panels, correspondingly. Arrows indicate the newborn granule cell that has a basal dendrite attached to the soma. ML, molecular layer; GCL, granule cell layer.

One microliter of retroviral CAG-GFP vector injected into the dentate gyrus labeled granule cells for approximately 3 mm in the septotemporal direction. More than 10 weeks after viral vector injection, GFP-expressing somata with processes could be seen in both the suprapyramidal and infrapyramidal blades of control or SE rats. These cells were scattered along the granule cell layer-hilus border (Figure 1). Occasionally, cells with a single basal dendrite that extended into the hilus were noticed; more often they were seen in SE rats.

### Dendritic complexity of mature granule cells born after status epilepticus

**Figure 2:**
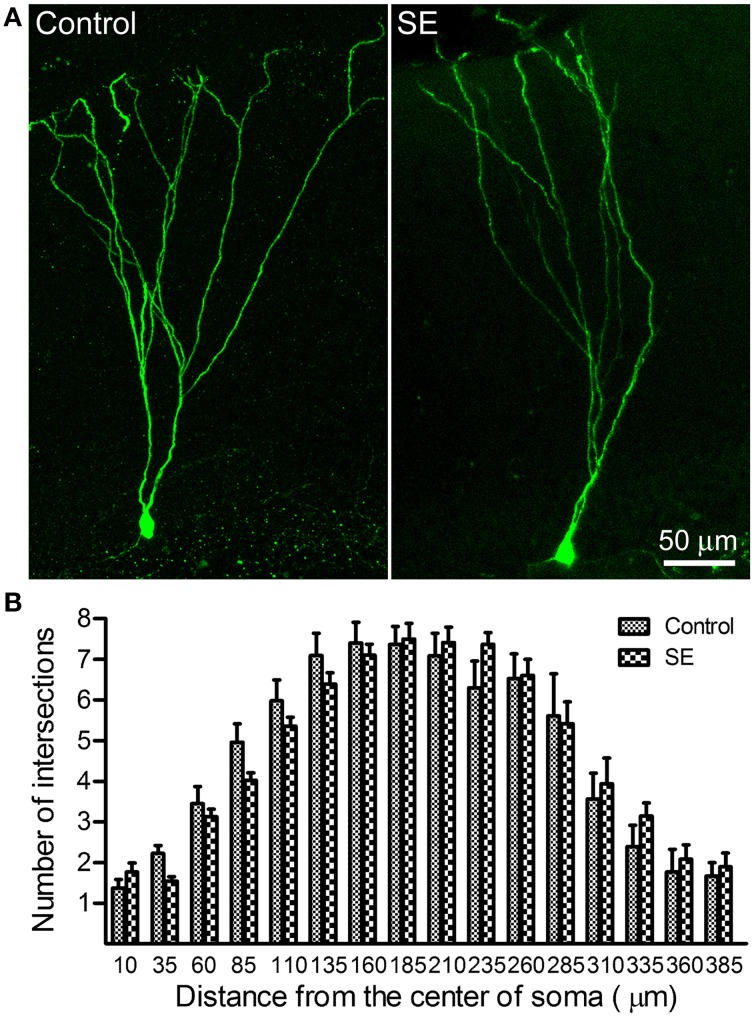
**Sholl analysis dedicates the dendritic complexity of mature newborn granule cells in control and SE rats**. Z-series stacks of 2 μm thick were taken in GFP-positive cells located in the suprapyramidal blade with a 20X objective (zoom = 1) and, thereafter, a 3D-image was created for each cell. After the soma and the apical dendritic arborizations were traced under ImageJ (http://imagej.nih.gov/ij/) with the NeuronJ plugin (http://www.imagescience.org/meijering/software/neuronj//), Sholl analysis was carried out by using ImageJ with the “Sholl analysis” plugin. **(A)** Representative images of retroviral labeling of newborn granule cells in a control (*left panel*) and an SE rat (*right panel*). Note that the granule cell born after SE has branch orders and arborizations similar to that of one born naturally. **(B**) Bar graph shows the dendritic complexity of newborn granule cells revealed by Sholl analysis. In both the control and SE groups (*n* = 8 animals each), 3–4 cells for each rat were scanned and all values at a given distance were averaged. Data are statistically compared using repeated-measure ANOVA.

Sholl analysis was used to determine the branching of apical dendrites. As shown in the left panel of Figure 2A, the apical dendrite of granule cells born in a control rat had 4–5 branch orders and their distal branches always reached the outer molecular layer. The apical dendrites of granule cells born after SE had very similar dendritic branch orders and arborizations also extended into the outer molecular layer (Figure 2A, right panel). Quantitative data were collected from eight rats in either control or SE group (Figure 2B); for each rat, 3–4 cells were scanned and the values are averaged to present the animal. Statistical comparisons done with repeated measure ANOVA revealed no statistical difference [*F*_(0.01, 1)_, *p* = 0.942] in dendritic branching between mature granule cells born after SE and those granule cells born naturally.

### A comparison of spine density in granule cells born after status epilepticus to those born naturally

**Figure 3:**
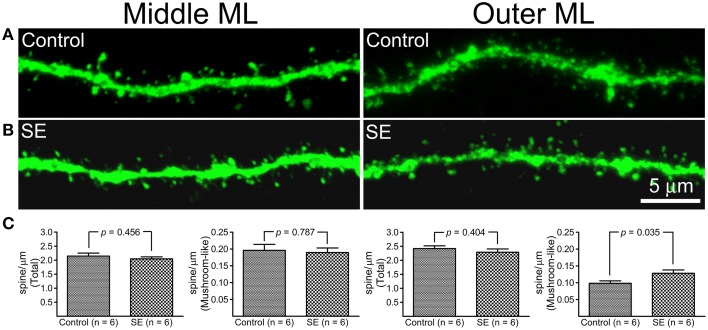
**A comparison of dendritic spine density in mature granule cells born after status epilepticus (SE) or sham treatment (Control)**. Z-series stacks were conducted in dendritic segments that were located in the middle molecular layer (*middle ML*) and outer molecular layer (*Outer ML*). **(A)** Representative confocal images of dendritic segments in the middle ML and outer ML in a control rat. **(B)** Representative confocal images of dendritic segments in the middle ML and outer ML in an SE rat. **(C)** Bar graphs comparing total and mushroom-like spine densities between the control and SE rats (*n* = 6 animals each). For each animal, spine densities were calculated from three cells; total six dendritic segments (three in the middle molecular layer and another three in the outer molecular layer) each cell were scanned and all values in the same layer were averaged. The left two panels represent dendritic segments in the middle molecular layer, and the two panels in the right represent dendritic segments in the outer molecular layer.

Z-series stacks were made in dendritic segments that were located in the middle and outer molecular layers and dendritic spines were counted on constructed 3D-images. Representative images and statistical comparisons are shown in Figure 3. Both mushroom-like and non-mushroom-like spines can be readily identified (Figures 3A,B). In the control rats, total spine densities in dendritic segments of the middle molecular and outer molecular layers were 2.15 ± 0.11 and 2.42 ± 0.09 spines/μm (*n* = 6), respectively. For the dendritic segments located in the middle molecular layer, both total and mushroom-like spine densities were not statistically different between the controls (*n* = 6) and SEs (*n* = 6) (Figure 3C, left two panels). However, mushroom-like spine density in the dendritic segments located in the outer molecular layer in SE rats (*n* = 6) was significantly denser than that in the control rats (*n* = 6), as shown in the right panel of Figure 3C.

### Intrinsic membrane properties and firing characteristics of mature granule cells born after status epilepticus

**Figure 4:**
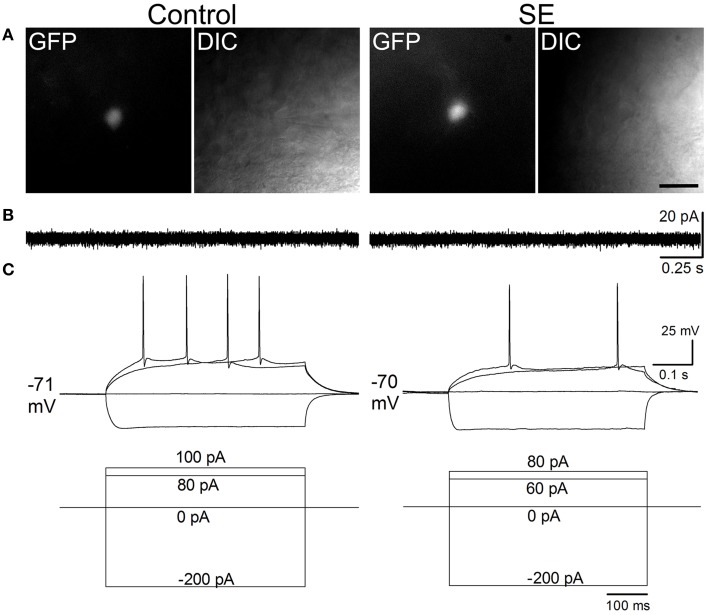
**Representative traces comparing spontaneous and evoked firings in mature granule cells of the control and SE groups**. The CAG-GFP retroviral vector was injected into the dentate gyrus 5 days after pilocarpine-induced SE or saline injection. Brain slices were prepared at least 10 weeks after retroviral vector injections. **(A)** Fluorescent (GFP) and differential interference contrast (DIC) images show recorded cells from a control (Control) and a rat suffering from SE before retroviral vector injection. **(B)** Spontaneous firing recorded in cell-attached mode. Note that no spontaneous firings were noticeable. **(C)** Evoked firing recorded in whole-cell mode. The current injection traces are shown beneath the membrane potential traces. Note that a doublet of bursting is seen in response to 70 pA current injection in the SE rat.

Figure 4 and Table 1 compare the intrinsic membrane properties and firing characteristics of mature granule cells born after SE to those granule cells born naturally. Epifluorescent and DIC images of the represented newborn granule cells are shown in Figure 4A. In cell-attached mode at near resting membrane potential, none of the newborn granule cells was found to have persistent spontaneous firing (Figure 4B); the percentages of newborn granule cells with occasional spontaneous firing are 11.8% (2/17) in the control group and 17.6% (3/17) in the SE group (*n* = 17) (*P* > 0.1, by Chi-square test). After membrane rupture, resting membrane potentials were −76.0±1.1 mV in the controls (*n* = 17), and −75.8 mV ± 0.9 mV in the SE rats (*n* = 17), respectively. Two cells in the control group (33%, *n* = 6) and 2 cells in SE group (33%, *n* = 6) displayed action potential doublets in response to stepped current injection at some current step (*P* > 0.1, by Chi-square test); however, none can be classified as burst firing because the interval between any two adjacent spikes was longer than 10 ms (Metz et al., 2005). The threshold potentials for firing were not significantly different between the control and SE groups (Table 1). Action potential parameters, including the amplitude, width, after depolarization (ADP), and area of ADP were not statistically different between the control (*n* = 6) and SE (*n* = 6) groups (Table 1).

**Table 1:** **A comparison of intrinsic membrane and firing properties of newborn granule cells in the control and SE groups**.

**Parameters**	**Control**	***SE***	***P*-value**
% of cells with spontaneous firing	11.8 (2/17)	17.6 (3/17)	>0.10^*^
Resting membrane potential (mV)	−76.0±1.1	−75.8±0.9	0.8476
Input resistance (MΩ)	211.6 ± 16.6	225.4 ± 20.6	0.6050
Membrane capacitance (mF)	35.1 ± 3.2	30.8 ± 4.2	0.4240
Membrane time constant	6.89 ± 0.5	6.0 ± 0.6	0.2521
Threshold potential (mV)	−48.0±2.4	−49.0±1.4	0.7362
Amplitude of AP (mV)	77.7 ± 6.6	83.1 ± 1.8	0.4154
Width of AP (ms)	1.6 ± 0.2	1.5 ± 0.1	0.7413
ADP (mV)	4.6 ± 1.6	4.7 ± 0.5	0.8976
Area of ADP (mV.ms)	93.3 ± 26.3	120.1 ± 19.7	0.4393

*Spontaneous firing, resting membrane potential, input resistance, membrane capacitance, membrane time constant are collected from 17 cells each in the control and SE groups. Parameters of action potential (AP) including the threshold potential, amplitude, width, after depolarization (ADP) and area of (ADP) calculated for 6 cells each in the control and SE groups. Data are expressed as mean ± SEM and compared with unpaired t-test except the percentages of cells with spontaneous firing and burst firing*.

* *The percentage of cells with spontaneous firing was compared by the chi-square test*.

### Miniature inhibitory and excitatory postsynaptic currents

**Figure 5:**
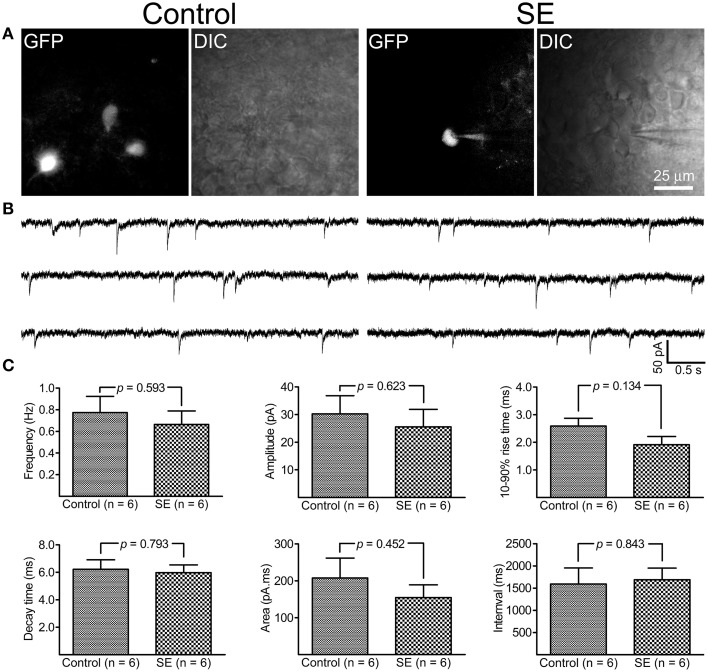
**Miniature inhibitory post-synaptic currents (mIPSCs) recorded from granule cells born after status epielpticus (SE) and sham treatment (Control)**. The retroviral CAG-GFP vector was injected into the dentate gyrus 5 days after induction of SE or saline injection and cells were recorded at least 70 days after retroviral vector injection. **(A)** Fluorescent (GFP) and differential interference contrast (DIC) images illustrate the recorded cells. **(B)** Representative traces of mIPSCs recorded at -70 mV at room temperature in the presence of 1 μM tetrodotoxin, 50 μM D-APV and 20 μM CNQX. **(C)** Quantitative comparisons of frequency, amplitude, 10–90% rise time, decay time, area, and interval time of mIPSC events. Statistical comparisons between the control (*n* = 6) and SE (*n* = 6) groups were made by unpaired *t*-test.

**Figure 6:**
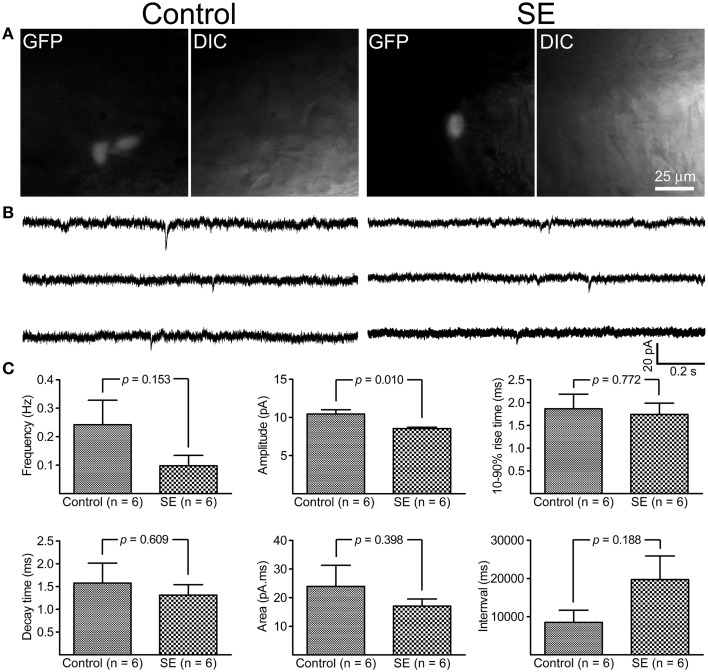
**Miniature excitatory post-synaptic currents (mEPSCs) recorded from granule cells born after status epilepticus (SE) and sham treatment (Control)**. The retroviral CAG-GFP vector was injected into the dentate gyrus 5 days after induction of SE or saline injection and cells were recorded at least 70 days after retroviral vector injection. **(A)** Fluorescent (GFP) and differential interference contrast (DIC) images illustrate the recorded cells. **(B)** Representative traces of mIPSCs recorded at -70 mV at room temperature in the presence of 1 μM tetrodotoxin- and 30 μM bicuculline. **(C)** Quantitative comparisons of frequency, amplitude, 10–90% rise time, decay time, area, and interval time of mEPSCs events. Statistical comparisons between the control (*n* = 6) and SE (*n* = 6) groups were made by unpaired *t*-test.

By holding cells at −70 mV, both mIPSCs and mEPSCs were recorded at room temperature. As shown in Figure 5, it appeared that mIPSCs recorded from newborn granule cells in the SE group were similar to those in the control group; statistical analyses revealed that neither frequency, amplitude, 10–90% rise time, decay time, area, nor interval time were significantly different between the control (*n* = 6) and SE (*n* = 6) groups. For mEPSCs (Figure 6), the amplitude in the SE group (*n* = 6) was significantly smaller than that in the control group (*n* = 6). In addition, there was a trend toward lower event frequency in the SE group (*p* = 0.153, control vs. SE).

### Cellular activity of mature granule cells born after status epileticus measured by arc expression in the resting condition or during the period of pentylenetetrazol (PTZ)-induced seizure

Specificity of rabbit anti-Arc used was tested in extracted rat hippocampal protein by immunoblot (Supplemental Figure 1). Arc could be immunofluorescently labeled in sections prepared from both the control and SE rats (Supplemental Figure 2). Co-localization experiment demonstrated that Arc was co-expressed with c-fos in the same cell with parallel intensity but different subcellular locations in the rest and during pilocarpine-induced SE (Supplemental Figure 3).

**Figure 7:**
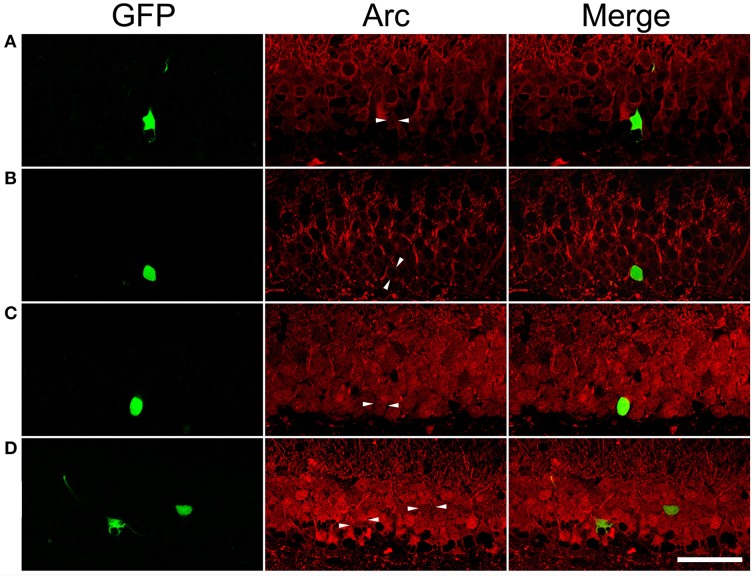
**Arc immunoreactivity in newborn granule cells of control and SE rats with or without pentylenetetrazol (PTZ) treatment**. Each GFP-labeled cell is indicated by two arrowheads in the middle panel. The scale bar (50 μm) applies to all panels. Note that Arc immunoreactivity in GFP-labeled cells is indistinguishable from surrounding granule cells in the same section. **(A)** In a PTZ-untreated control rat, Arc immunoreactivity is faint in the soma and dendrites of granule cells. **(B)** In a PTZ-untreated SE rat, Arc immunoreactivity became denser in the soma and apical dendrites of granule cells than that in PTZ-untreated controls, but the intensity of Arc immunoreactivity in the GFP-labeled cell is clearly indifferent from cells surrounding it. **(C)** Treatment with PTZ in a control rat increased Arc immunoreactivity in both the soma and apical dendrites of granule cells, including the GFP-labeled one. **(D)** Treatment with PTZ in an SE rat also increased Arc immunoreactivity in granule cells. However, Arc immunoreactivity in the GFP-labeled cell is similar to the vicinal granule cells unlabeled by GFP.

**Figure 8:**
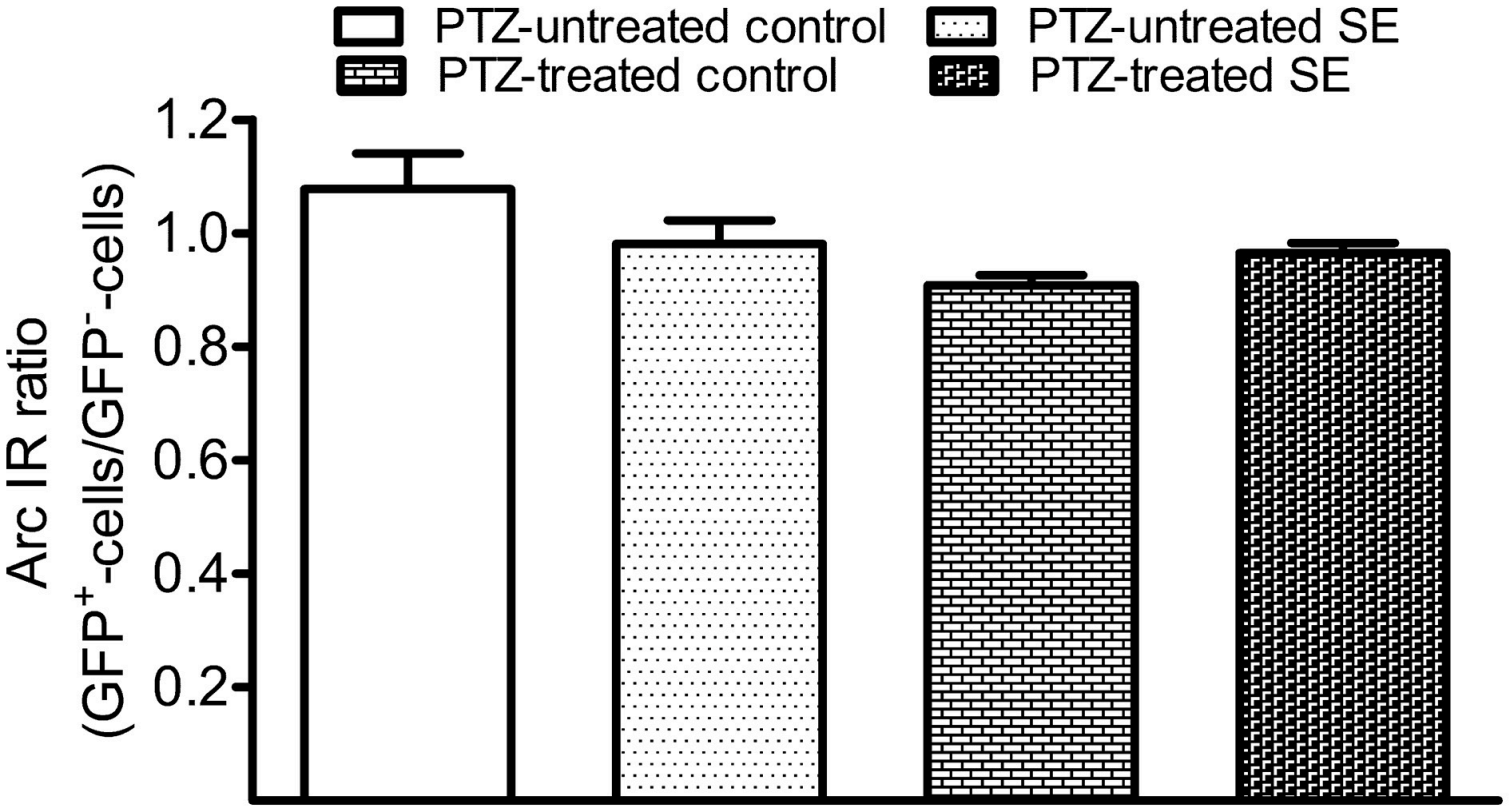
**Bar graphs compare Arc immunoreactivity (IR) in the GFP-labeled newborn granule cells to surrounding GFP-negative granule cells in the resting condition and during the period of a transient seizure**. The relative intensity of Arc IR in individual GFP-positive cells was normalized to the averaged Arc IR of 10 surrounding granule cells that represent existing granule cells. Each group contains six datasets in which each dataset was acquired from 10 GFP-labeled cells of each animal. Bars are standard error of the mean (SEM). Note that the Arc ratio is higher and more variable in PTZ-untreated control but very close to 1.0 in the rest of three groups. Statistical significances do not exist among the four groups as revealed by one-way variance analysis.

After PTZ treatments, tonic-clonic seizures were observed within 2 min in most rats and the intervals between two adjacent clonic seizures got longer over time. We terminated motor seizures by intraperitoneal injection of diazepam 15 min after the appearance of the first motor seizure. In PTZ-untreated controls, dentate granule cells constitutively expressed Arc at low level (Figure 7A). Arc immunoreactivity in granule cells of PTZ-untreated SE animals appeared more intense than that of granule cells in PTZ-untreated controls; however, the intensity of Arc immunoreactivity in the GFP-labeled newborn cells did not exceed that of nearby GFP-negative granule cells (Figure 7B). Treatment of controls with PTZ dramatically enhanced Arc immunoreactivity in the soma and apical dendrites of almost every granule cell (Figure 7C), but again, a difference between GFP-labeled granule cells and granule cells surrounding them could not be noticed. Treatment of SE rats with PTZ also increased Arc immunoreactivity in both the soma and apical dendrites of almost all granule cells and it appeared that the intensity of Arc immunoreactivity in GFP-labeled newborn granule cells was similar to that in adjacent GFP-negative granule cells (Figure 7D). Quantitative analyses that compared Arc immunoreactivity in GFP-labeled cells to the averaged Arc immunoreactivity of surrounding granule cells clearly shows that the intensities of Arc immunoreactivity in GFP-positive somata and dendrites were not different from surrounding granule cells in the resting condition (Figures 7A,B, 8) or during a transient seizure episode (Figures 7C,D, 8). Similar results were observed when c-fos was used as a cellular activity marker (Supplemental Figures 4, 5).

## Discussion

This study identified two significant differences between normotopic dentate granule cells born after SE or in a normal environment: in granule cells born after SE, the distal dendritic segments had a higher density of mushroom spines but the mean mEPSC amplitude was smaller. Otherwise, intrinsic passive and active membrane properties, inhibitory synaptic events, dendritic morphology, baseline activity, and response to PTZ seizures were indistinguishable.

In a number of epilepsy models, seizure activity, especially SE, is capable of enhancing hippocampal dentate neurogenesis (Bengzon et al., 1997; Parent et al., 1997; Scott et al., 1998; Nakagawa et al., 2000; Sankar et al., 2000; Scharfman et al., 2000; Jiang et al., 2003; Jiruska et al., 2013). According to the soma positioning, granule cells born after SE can be classified into normotopic and ectopic. Most ectopically located granule cells have been found in the hilus (hilar ectopic granule cells). A small number are also present in the molecular layer (Liang et al., 2013). Hilar ectopic granule cells are morphologically and electrophysiologically different from normotopic granule cells; specifically, when compared to normotopic granule cells, higher percentages of hilar ectopic granule cells have a basal dendrite extending into the hilus (Ribak et al., 2000; Shapiro et al., 2005; Cameron et al., 2011), exhibit spontaneous firing and/or generate burst firing in response to depolarization (Scharfman et al., 2000; Zhan and Nadler, 2009; Althaus et al., 2015), and demonstrate unbalanced excitation and inhibition (Dashtipour et al., 2001; Zhan et al., 2010). Because of their specific intrinsic membrane properties and imbalanced excitation and inhibition, hilar ectopic granule cells are considered to contribute to the development of spontaneous seizures or to the propagation of epileptic waveforms (for reviews see Parent et al., 2006; Scharfman and Pierce, 2012; Myers et al., 2013). Normotopic granule cells born following SE far outnumber hilar ectopic granule cells (Parent et al., 1997). However, there is no consensus on the role of normotopic granule cells in the development of epilepsy (Bielefeld et al., 2014). Morphological studies conducted on normotopic granule cells born after SE have found some dendritic abnormalities in comparison with those being generated in physiological conditions. First, a higher percentage of granule cells born after status SE was found to have a basal dendrite that extends to the hilus (Ribak et al., 2000; Shapiro et al., 2005; Avanzi et al., 2010; Cameron et al., 2011). Second, the soma sizes of granule cells born after SE vary considerably (Murphy et al., 2011). Finally, abnormal dendritic morphologies of newborn granule cells have been noticed in some epileptic models (Murphy et al., 2012). These findings indicate that newborn granule cells may function differently from those born in physiological conditions and, as a consequence, contribute to the development of epilepsy. A recent study has concluded that the accumulation of abnormal adult-generated hippocampal granule cells positively correlates with seizure frequency and severity in mice (Hester and Danzer, 2013). However, several pieces of evidence contradict the role of transient up-regulation of neurogenesis in the development of epilepsy. First, increased neurogenesis did not occur after status epilepticus in aged rats despite the eventual development of spontaneous seizures (Rao et al., 2008). Second, most studies have been conducted in immature granule cells following status epileticus instead of mature adult-born granule cells (Arisi and Garcia-Cairasco, 2007; Murphy et al., 2012; Hester and Danzer, 2013); many immature granule cells die in the process of maturation (Bengzon et al., 1997; Ekdahl et al., 2001, 2003), especially those with abnormal morphology, and often behave more like preexisting granule cells after maturation (Walter et al., 2007). In addition, synaptic activity recordings illustrated that newborn granule cells are hypoactive instead of hyperactive (Jakubs et al., 2006; Wood et al., 2011). Finally, in different epilepsy models, elimination of neurogenesis with irradiation following SE was found to be ineffective in suppressing the development of spontaneous seizures (Parent et al., 1999; Pekcec et al., 2011).

Our study focused mature newborn granule cells instead of developing adult-born granule cells because neurogenesis after SE is enhanced only transiently. Previous studies have shown that, upon maturation, granule cells born naturally have morphological and electrophysiological properties similar to those of preexisting granule cells (Laplagne et al., 2006, 2007; Zhao et al., 2006; Morgenstern et al., 2008; Toni et al., 2008). Our results suggest that, when mature, granule cells born after SE are normally active or less active than those granule cells born in a normal environment.

The geometry of dendritic braches and the distribution of synapses play important roles for neurons in receiving and integrating signals. In comparison with other types of neurons such as pyramidal cells, hippocampal dentate granule cells require considerably more concurrent inputs to generate an output (Krueppel et al., 2011). A minor change in the dendritic morphology of granule cells probably would not affect their output significantly. In the present study, Sholl analysis revealed that the dendritic branching of mature granule cells born after SE was similar to that of granule cells born in age-matched control rats. Dendritic spines are considered to be the sites where most excitatory synapses are formed. Perforant path axons arising in layers II of the entorhinal cortex synapse onto the dendritic spines of granule cells. In the control rats, the total spine densities of mature granule cells counted in dendritic segments located in the middle and outer molecular layers were approximately 2.2 and 2.4 spines/μm, respectively, with the mushroom-like spines accounting for about 10% of total spines. These numbers are nearly identical to densities reported by other investigations (Laplagne et al., 2007; Zhao et al., 2014). Compared to granule cells born in physiological conditions, spine density in granule cells born after SE was similar in the dendritic segments of the middle molecular layer but was significantly higher in the dendritic segments of the outer molecular layer. This may reflect that synapses formed on the dendritic segments in the middle and outer molecular layers are differentially regulated by entorhinal inputs. After generation, the peak of spine growth in newborn granule cells occurs between 2 and 4 weeks (Schmidt-Hieber et al., 2004) and the number of mushroom-like spines starts to increase after this time until complete maturation of the cell (Zhao et al., 2006; Toni et al., 2008). The growth of mushroom-like spines is regulated by living environments that determine presynaptic inputs onto newborn granule cells (Zhao et al., 2006, 2014). Perforant path axons forming synapses onto granule cells are topologically arranged; stellate cells in layer II of lateral and medial entorhinal cortex project to the outer and middle molecular layers, respectively (Canto et al., 2008). Grid cells, boundary cells, and head-direction cells reside in the medial entorhinal cortex and provide spatial information to the dentate gyrus, whereas neurons in the lateral entorhinal cortex respond to stimuli such as odor and objects and provide non-spatial information to the dentate gyrus (Fyhn et al., 2004). In animal models of temporal lobe epilepsy, stellate cells generate excessive, spontaneous, and hypersynchronous input to dentate granule cells once stimulated (Kobayashi et al., 2003; Kumar et al., 2007). It is possible that the increased non-spatial output from the entorhinal cortex in animals with spontaneous seizures might have been responsible for the higher mushroom-like spine density in the outer molecular layer observed in the present study. Indeed, after pilocarpine-induced status epilepticus, preexisting granule cells have been found to have more distal dendritic branches (Cameron et al., 2011). Regarding how spontaneous seizures would affect the development of newborn granule cells, an earlier study found that spontaneous seizures promoted the maturation of newborn granule cells (Overstreet-Wadiche et al., 2006). Our spine counting does agree with their observation. However, a later study done in mice by Shin and colleagues found that pilocarpine-induced SE was associated with an increased expression of calretinin but a decreased expression of calbindin in couple with granule cells becoming more depolarizing, implicating that granule cells entered into a de-maturation mode after SE (Shin et al., 2013). We also observed a dramatic down-regulation of calbindin expression by granule cells after SE, however, quantitative analysis is needed to know if difference exists between those preexisting granule cells and mature newborn granule cells (Supplemental Figure 6).

The intrinsic membrane properties of mature granule cells born after SE are not different much from those granule cells born in normal environment; this is generally in agreement with a recent rat study in which granule cells were recorded between 2 and 4 months after birth (Althaus et al., 2015). As the intrinsic membrane properties of mature newborn granule cells born after SE are similar to those born naturally, one can speculate that similar dendritic arborization and spine density would predict that the synaptic activity of granule cells born after SE would be similar to granule cells born naturally. Indeed, the only difference in synaptic activity observed between the control and SE rats was that mature granule cells born after SE has smaller amplitude of mEPSCs in addition to a tendency of fewer mEPSC events. The lesser frequency might have been due to the smaller events, which certainly would make a portion of smaller current events undetectable. This suggests that excitatory synapses formed on newborn granule cells after SE may be hypofunctional, as shown by previous studies (Jakubs et al., 2006; Wood et al., 2011). As the mushroom-like spine density was higher in the dendritic segments locating in the outer molecular layer of SE rats, it is puzzling that less excitatory inputs were received. Nevertheless, the weaker excitatory synaptic response observed in mature newborn granule cells after SE corresponded with a lower Arc expression in the rest condition and during PTZ-induced seizure activity. As the amplitude of mEPSCs is determined by not only the number of excitatory synapses but also the density and subunit composition of glutamate receptor in the synaptic sites, the smaller amplitude of mEPSCs in newborn granule cells after SE might have been caused by abnormal expression of glutamate receptors. It is known that granule cells display a maturational shift from NMDA-dominated to AMPA-dominated glutamatergic transmission (Ye et al., 2005). AMPA receptors form tetramers that are assembled as combinations of the subunits GluR1-4. While it has not been studied in newborn granule cells in the model of epilepsy, a study performed in an animal model of schizophrenia and bipolar disorder showed that both GluR1 and GluR2 immunoreactivities were substantially down-regulated in 3–4 week old dentate granule cells (Hagihara et al., 2011). The weaker excitatory neurotransmission may underlie cognitive impairment and other behavioral disturbances seen in animal models of epilepsy (Chauvière et al., 2009; Cavarsan et al., 2013).

Since it was possible that only very healthy GFP-labeled granule cells after SE could have been patched and recorded, we attempted to compare the cellular activity of mature granule cells after SE in the resting condition and during a period of transient seizure episode by analyzing Arc expression. Arc is one of the immediate early genes that plays a crucial role in activity-dependent synaptic plasticity and consolidation of long-term memory (Guzowski et al., 2005; Shepherd and Bear, 2011). After intense neuronal activity, Arc mRNA accumulates in the nucleus within minutes, translocates into the cytoplasm a half hour later, and is then degraded rapidly after behavioral stimuli. The appearance of newly synthesized Arc protein in the cytoplasm and dendrites parallels the increased Arc mRNA with little time delay (Guzowski et al., 2005; Ramírez-Amaya et al., 2005; Shepherd and Bear, 2011; Soulé et al., 2012). Although concern was raised in using Arc to indicate the activation of young granule cells (Matsuo et al., 2009), it should not possess any problem for granule cells more than 10 weeks old, because a study already demonstrated that granule cells would have full capacity in expressing Arc 5 weeks after birth (Jungenitz et al., 2013).

Using Arc immunoreactivity as an indicator of granule cell activation, we found that granule cells born after pilocarpine-induced SE did not express Arc more intensely than the surrounding granule cells and, in addition, transient seizure activity induced by pentylenetetrazol did not activate mature granule cells born after SE more intensely. The tendency of lower activity in newborn granule cells after SE likely relates to the reduced excitatory neurotransmission, suggesting that mature granule cells born after SE do not contribute to dentate network hyperactivity. As transplantation of neural stem cells into the hippocampus significantly improves cognitive function and other behavioral abnormalities in different epilepsy models (Shetty, 2014) and integration of newborn granule cells is important for cognitive function in a kindling model (Fournier et al., 2013), a careful evaluation of the significance of transiently increased neurongenesis after SE is needed.

In the rat pilocarpine model of temporal lobe epilepsy, most animals develop spontaneous seizures with similar frequency and severity regardless of the severity of SE (Cavalheiro et al., 1987; Goffin et al., 2007). Despite this, both short- and long-term pathological changes vary according to the severity and duration of SE (Jafari et al., 2012). For example, severe SE led to more robust granule cell proliferation but increased death of newborn granule cells, whereas mild status epilepticus brought less robust neurogenesis but higher survival of newborn granule cells (Mohapel et al., 2004). Factors such as the age of cells, the severity of SE and the frequency of spontaneous seizures might have contributed to some differences observed in the present study and previously published ones. It appears that the most important contributor could be the age of newborn granule cells. Take the percentage of newborn granule cells with basal dendrite as an example, a previous study has documented that mature newborn granule cells with basal dendrite after SE became less frequent than those immature ones and biased toward to granule cells born naturally (Walter et al., 2007).

In conclusion, upon maturation, granule cells born after pilocarpine-induced status epilepticus do not appear hyperactive in comparison with those born in physiological conditions. Without continuous abnormal neurogenesis, the transient enhancement of neurogenesis would not sustain hyperactivity.

### Conflict of interest statement

The authors declare that the research was conducted in the absence of any commercial or financial relationships that could be construed as a potential conflict of interest.
