# Supplementary material for: Dendritic morphology, synaptic transmission, and activity of mature granule cells born following pilocarpine-induced status epilepticus in the rat
Source: Front Cell Neurosci. 2015 Oct 7;9:384. doi: 10.3389/fncel.2015.00384 (PMC4596052; doi:10.3389/fncel.2015.00384)
Supplement: Supplementary file 1 [file DataSheet1.PDF]

Supplementary Material

Dendritic morphology, synaptic transmission, and activity of mature granule cells born following pilocarpine-induced status epilepticus in the rat

Fei Gao[1], Xueying Song[1], Dexiao Zhu[1], Xiaochen Wang[1], Aijun Hao[2], J Victor Nadler[3], Ren-Zhi Zhan[1*]

[1]Department of Physiology, Shandong University School of Medicine, Jinan, Shandong Province, China
[2]Department of Histology and Embryology, Shandong University School of Medicine, Jinan, Shandong Province, China
[3]Departments of Pharmacology and Neurobiology, Duke University Medical Center, Durham, NC, USA

***Correspondence:** Dr. Ren-Zhi Zhan, Department of Physiology, Shandong University School of Medicine, Jinan 250012, China

1. Supplemental Fig. 1.

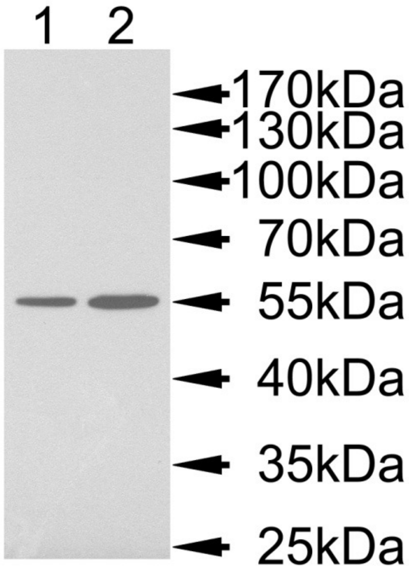

Fig.1. Specificity of rabbit anti-Arc determined by immunoblot

Proteins extracted from rat hippocampi were separated by electrophoresis, and then transferred onto nitrocellulose membranes. Application of a rabbit anti-Arc in a dilution of 1:10000 yielded single band which is at ~55 kDa. In lanes 1 and 2, the amounts of proteins loaded were 10 and 20 μg, respectively.

2. Supplemental Fig. 2.

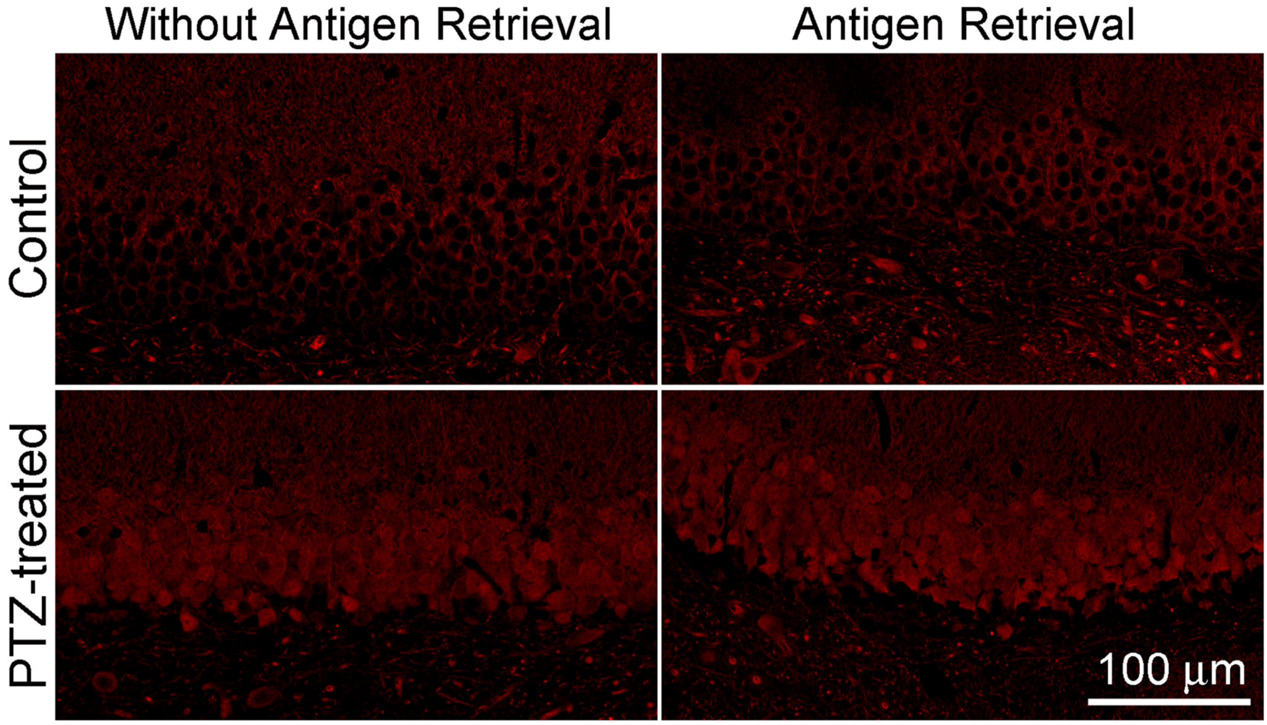

Fig. 2. Arc labeling is not improved by an antigen retrieval procedure

Seizures were induced by an intraperitoneal injection of pentylenetetrazole (PTZ) (20 mg/kg) in male Sprague-Dawley rats more than 18 weeks old and terminated 15 min later by an intraperitoneal injection of diazepam (5 mg/kg). The controlled animal received equivalent volume of normal saline as the PTZ replacement and diazepam (5 mg/kg) with the same time schedule. Rats were transcardially perfused with 4% paraformaldehyde buffered with 0.1 M PBS 2 h after seizure termination. After an overnight post-fixation and subsequently immersed in gradually increased concentration of sucrose in 1XPBS, coronal hippocampal sections were cut into 40 μm in thickness with a vibratome. Sections from both the controlled (control) and PTZ-treated (PTZ-treated) animals were stained with a rabbit anti-Arc (Synaptic System, Goettingen, Germany; 1:1000 dilution) with or without antigen retrieval procedure.

Antigen retrieval procedure was done by incubating sections in a 20 mM sodium citrate solution buffer (pH 8.8) preheated to and maintained at 80°C in a water bath for 30 min (Jiao et al., 1999). The stained sections were scanned with a Zeiss LSM 780 (X20 objective and 1.0 zoom).

3. Supplemental Fig. 3.

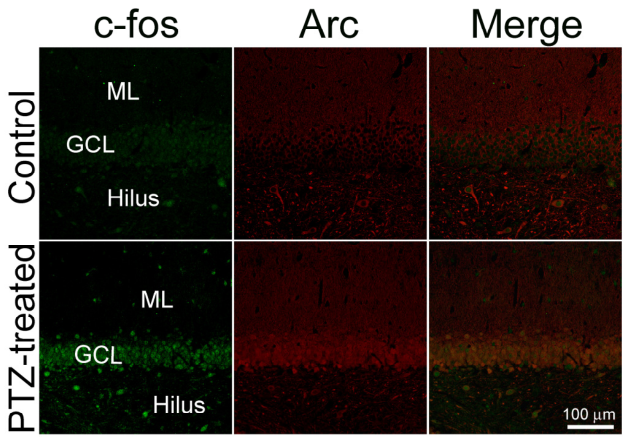

Fig. 3. Parallel expression of Arc and c-fos in granule cells in the rest and after PTZ-induced transient seizure

The induction of seizures and tissue preparation were described in the supplemental Fig. 2. Sections were double-stained with a goat anti-c-fos (1:2000, Abcam, Cambridge, UK) and a rabbit anti-Arc (1:1000, Synaptic System, Goettingen, Germany). The secondary antibodies used were Alexa Flour 488-conjugated donkey anti-goat IgG and Alexa Flour 568-conjugated donkey anti-rabbit IgG (1:600, Invitrogen, Carlsbad, CA, USA).

Note that the expression of Arc is parallel to that of c-fos in term of intensity but different subcellular locations in the control (upper row) and after PTZ treatment (PTZ-treated). Noticeably, c-fos expression was seen in some glia-like cells in addition to neuronal expression. ML = Molecular layer; GCL = granule cell layer.

4. Supplemental Fig. 4.

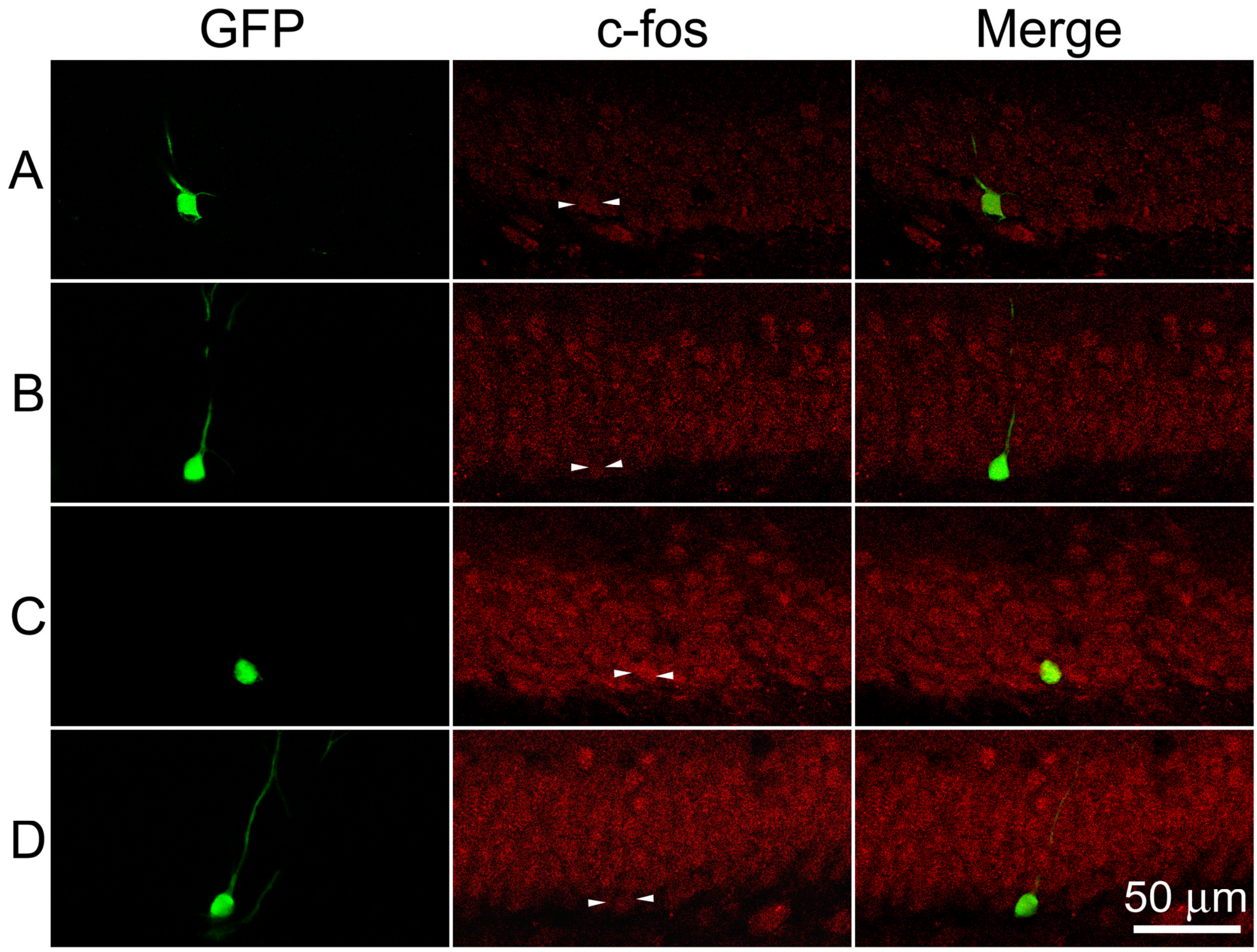

Fig. 4. c-fos expression in newborn granule cells of control and SE rats with or without pentylenetetrazol (PTZ) treatment

SE was induced by an intraperitoneal injection of pilocapine. At the 5th day after induction of SE or sham-treatment (control), 1 µl of CAG-GFP retroviral vector was injected into the dentate gyrus. More than 70 days after viral vector injection, animals were grouped and treated with PTZ (20 mg/kg) by a procedure that was identical for Arc staining. Hippocampal sections (40 µm in thickness) with the presence of at least one GFP-labeled granule cell were stained with a goat anti-c-fos (1:2000, Abcam, Cambridge, UK). Alexa Flour 568-conjugated donkey anti-goat IgG (Invitrogen, Carlsbad, CA, USA) was used as a secondary antibody (1:600). Sections were scanned by using a Zeiss LSM 780 confocal microscope equipped with a 40× objective (zoom 1). Each GFP-labeled cell is indicated by two arrowheads in the middle panel. The scale bar (50 µm) applies to all panels.

A: In a PTZ-untreated control rat, the whole population of granule cells shows faint c-fos immunoreactivity, however, the GFP-labeled cell has denser stain than adjacent granule cells.

B: In a PTZ-untreated SE rat, c-fos immunoreactivity became denser in the granule cells in comparison with those in PTZ-untreated controls. It appears that the intensity of c-fos immunoreactivity in the GFP-labeled cell is not different from granule cells surrounding it.

C: In a PTZ-treated control rat, c-fos immunoreactivity in granule cells is denser than that in either PTZ-untreated SE rat or the PTZ-untreated control rat. The intensity of c-fos immunoreactivity in the indicated GFP-labeled cell looks slightly denser than those GFP-negative granule cells.
D. In a PTZ-treated SE rat, c-fos immunoreactivity is increased to a degree as seen in "C". c-fos immunoreactivity in the GFP-labeled cell appears faint in comparison to vicinal granule cells.

5. Supplemental Fig. 5.

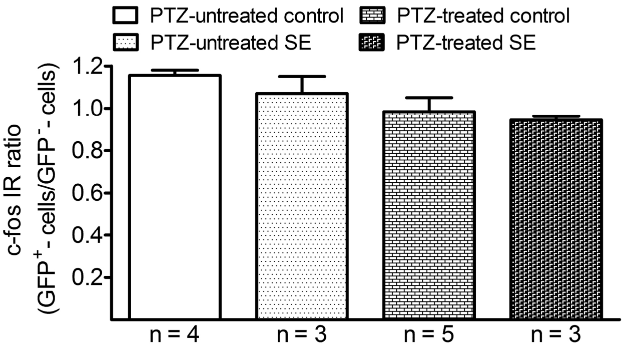

Fig. 5. Bar graphs compare c-fos immunoreactivity (IR) in the GFP-labeled newborn granule cells to surrounding GFP-negative granule cells in the resting condition and during the period of a transient seizure
The relative intensity of c-fos IR in individual GFP-positive cells was normalized to the averaged c-fos IR of 10 surrounding granule cells. In each animal, the ratios yielded from different GFP-positive cells are averaged. The number of animals examined is indicated under the X-axis. One-way ANOVA did not reveal statistical significance among the four groups [$F(3,15)$, $p = 0.0988$].

6. Supplemental Fig. 6.

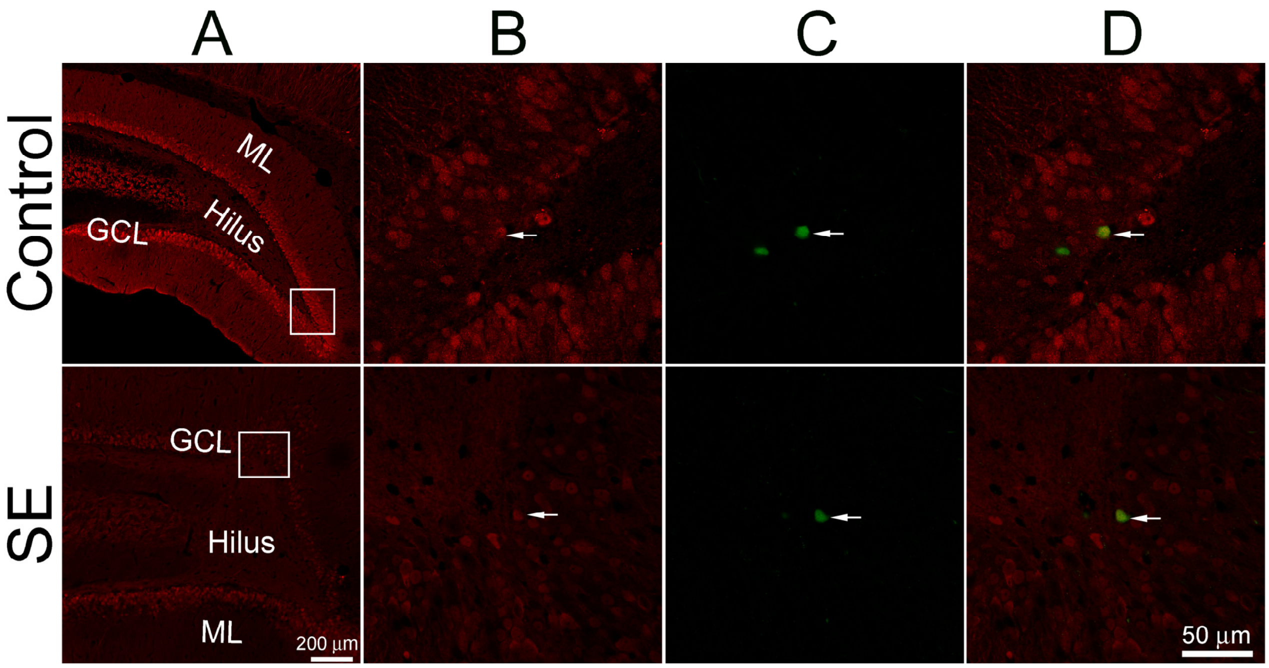

Fig. 6. Calbindin (CB) expression is indifferent between newborn and preexisting granule cells after pilocapine-induced SE

SE was induced by an intraperitoneal injection of pilocapine. Five days later, CAG-GFP retroviral vector was injected into the dentate gyrus. Transcardiac perfusion and tissue preparation were described in supplemental Fig. 4. Sections with GFP-labeled cells were stained with a rabbit anti-calbindin (1:4000, EMD Millipore, Billerica, MA, USA). The secondary antibody used was Alexa Flour 568-conjugated donkey anti-rabbit IgG (1:600, Invitrogen, Carlsbad, CA, USA). The stained sections were scanned with a Zeiss LSM 780 (X10 and X40 objectives, zoom 1.0).

A: Images of CB were taken by using an X10 objective.

B and C: The area indicated by the white box was imaged by using an X40 objective for CB (B) and GFP (C).

D: Merged “B” and “C”.

ML = molecular layer; GCL = granule cell layer

Newborn granule cells are indicated by arrows.

References

Jiao, Y., Sun, Z., Lee, T., Fusco, F.R., Kimble, T.D., Meade, C.A., Cuthbertson, S., Reiner, A., (1999). A simple and sensitive antigen retrieval method for free-floating and slide-mounted tissue sections. J. Neurosci. Methods. 93, 149-162.
