# Supplementary figures and images for: Dendritic morphology, synaptic transmission, and activity of mature granule cells born following pilocarpine-induced status epilepticus in the rat
Source: Front Cell Neurosci. 2015 Oct 7;9:384. doi: 10.3389/fncel.2015.00384 (PMC4596052; doi:10.3389/fncel.2015.00384)

PTZ-untreated control

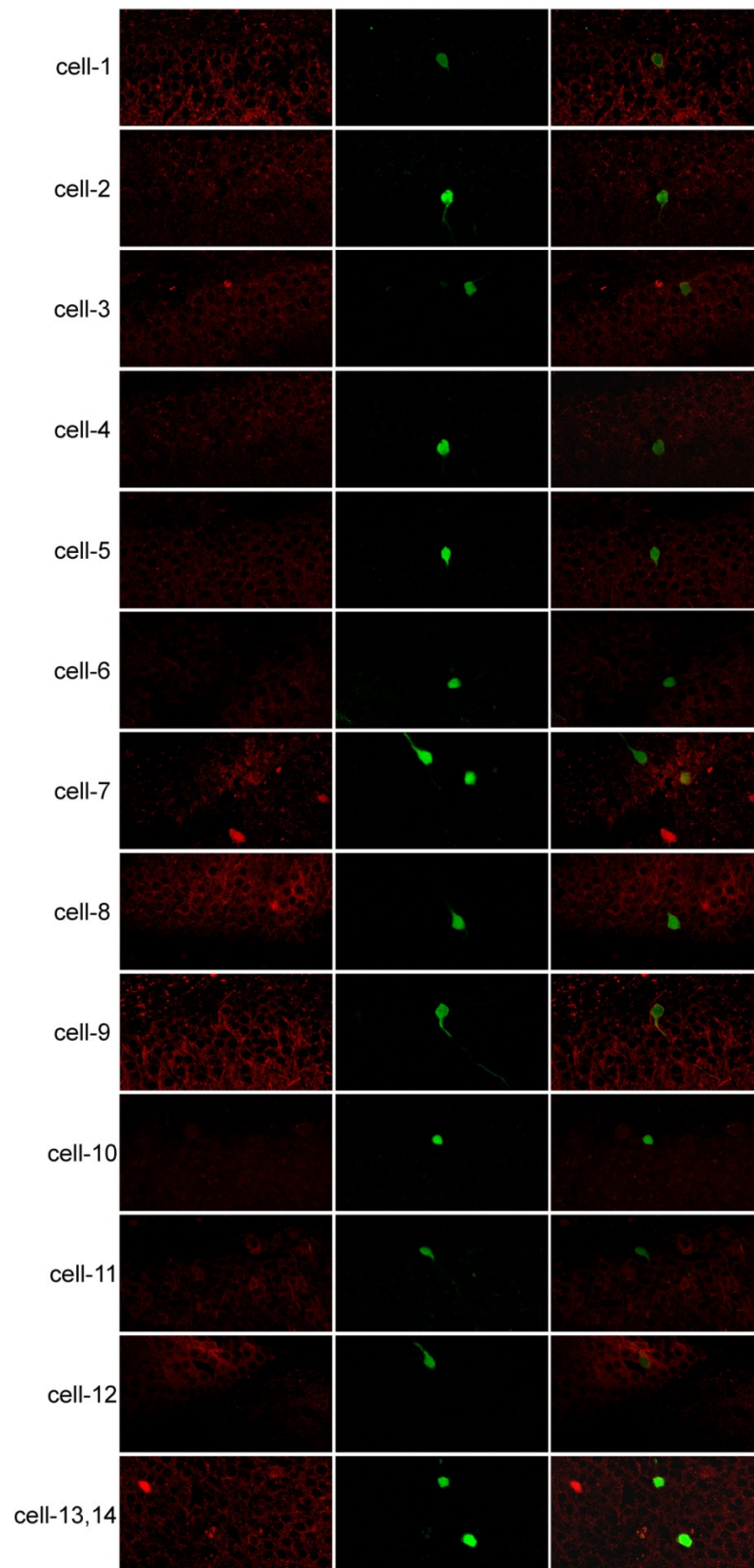

PTZ-untreated control

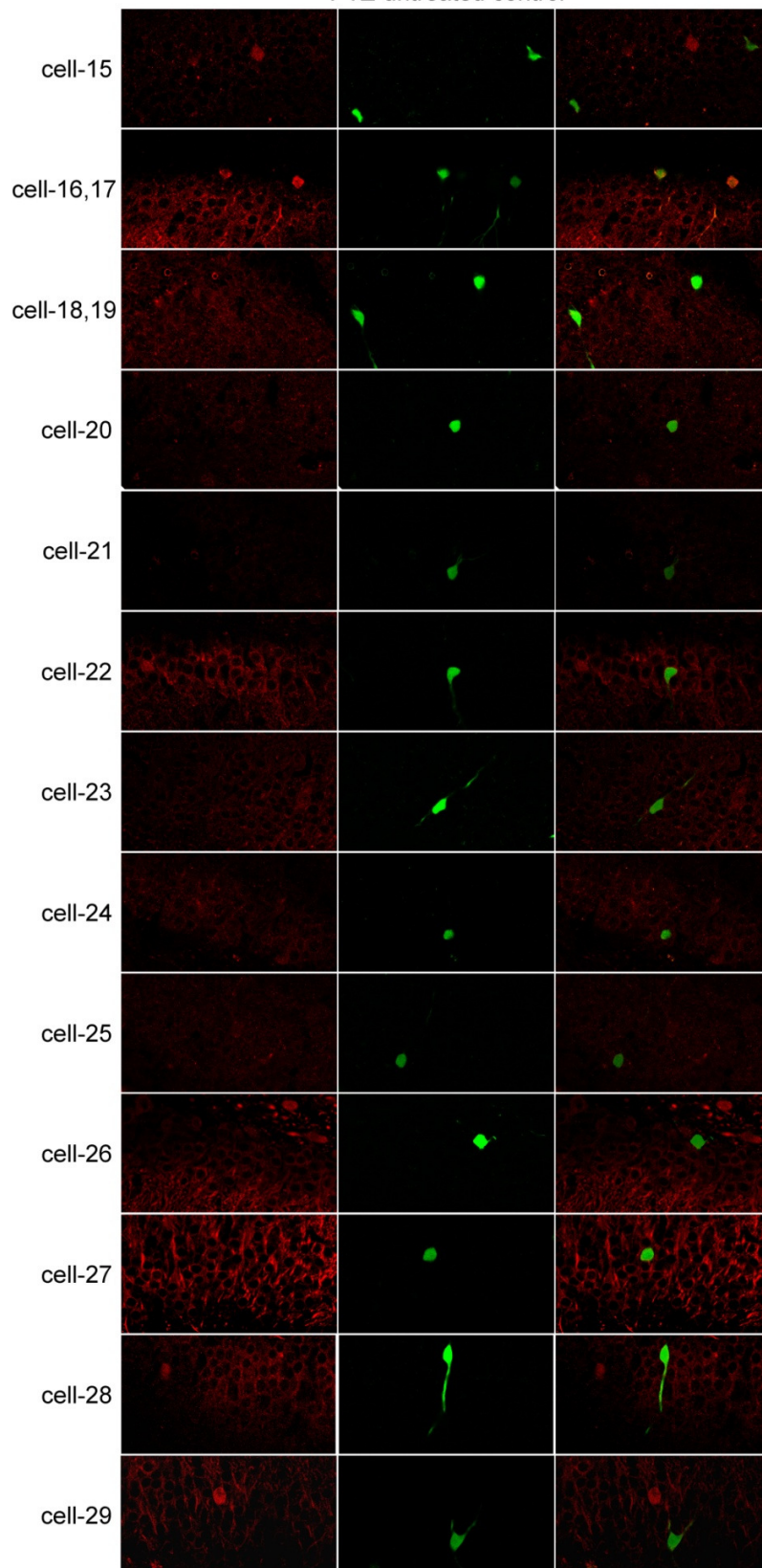

PTZ-untreated control

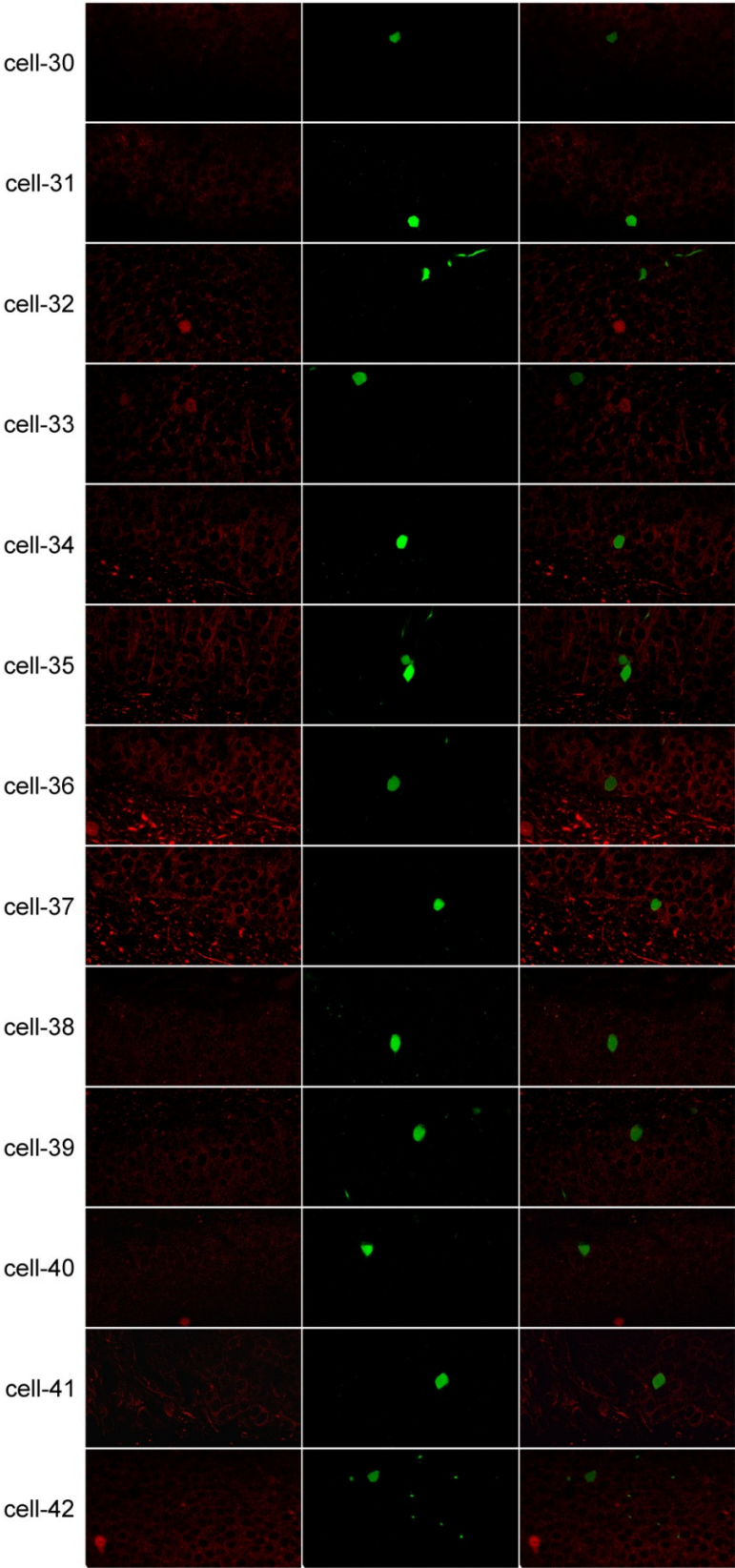

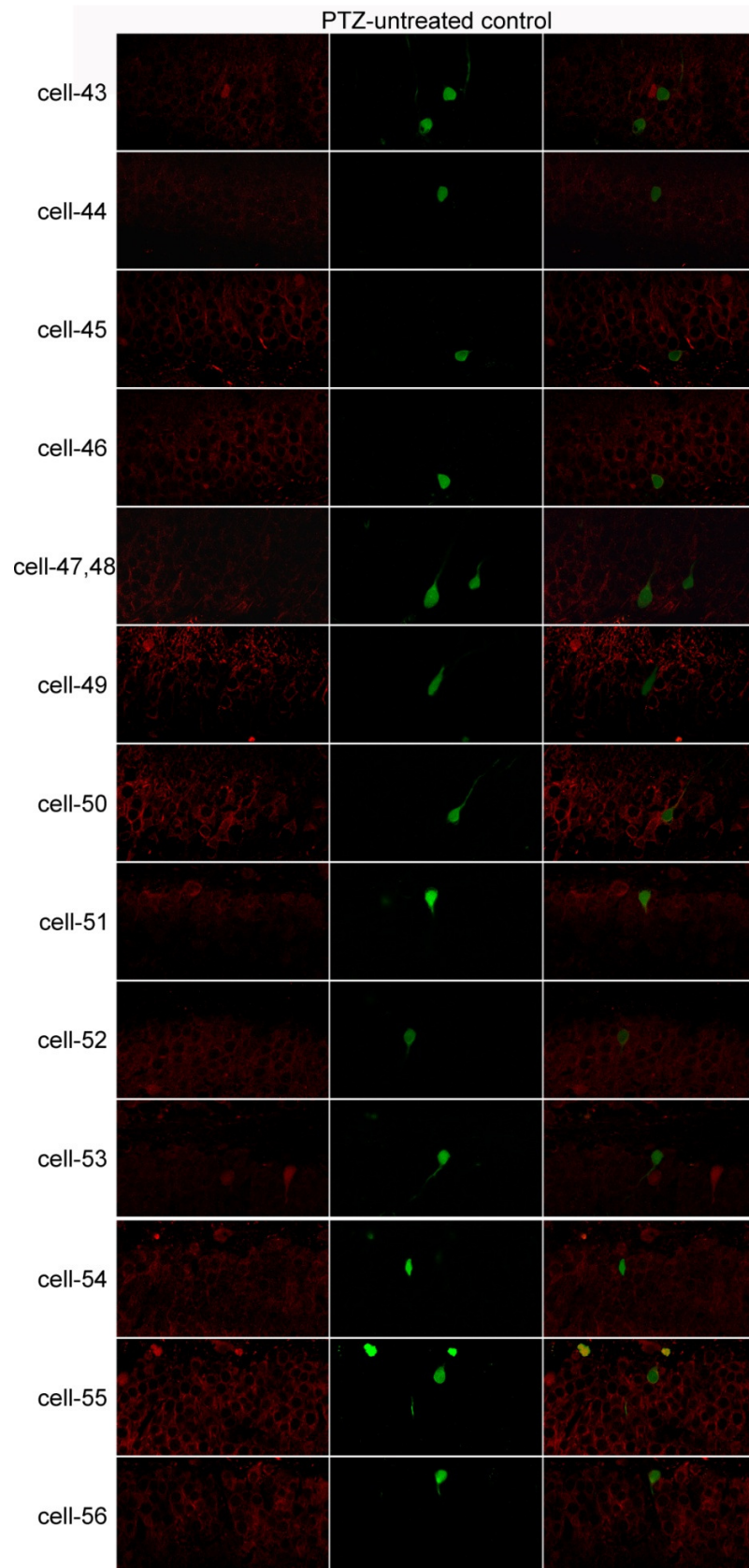

PTZ-untreated control

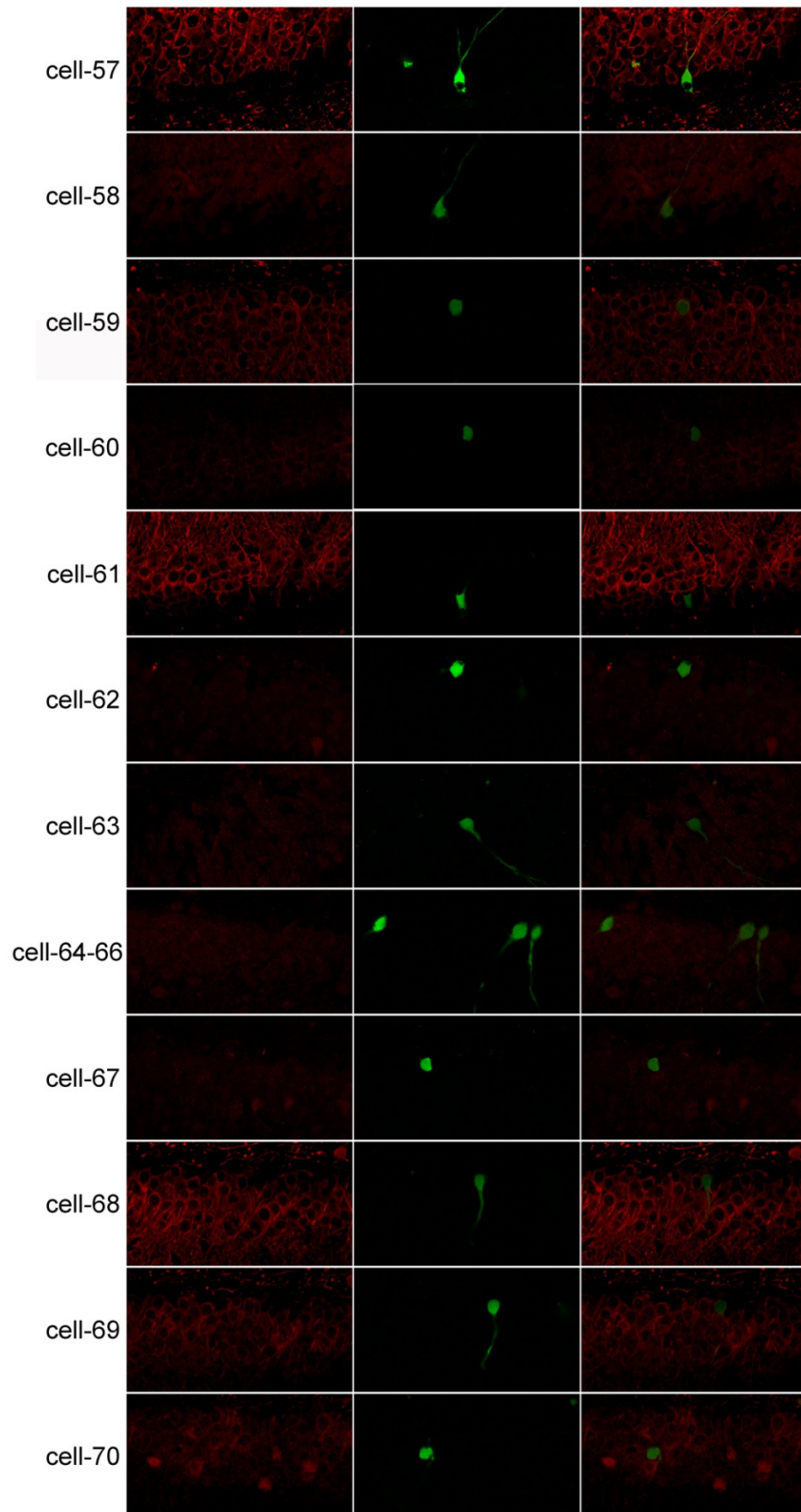

Supplement: Supplementary file 2 [file DataSheet2.PDF]

PTZ-untreated SE

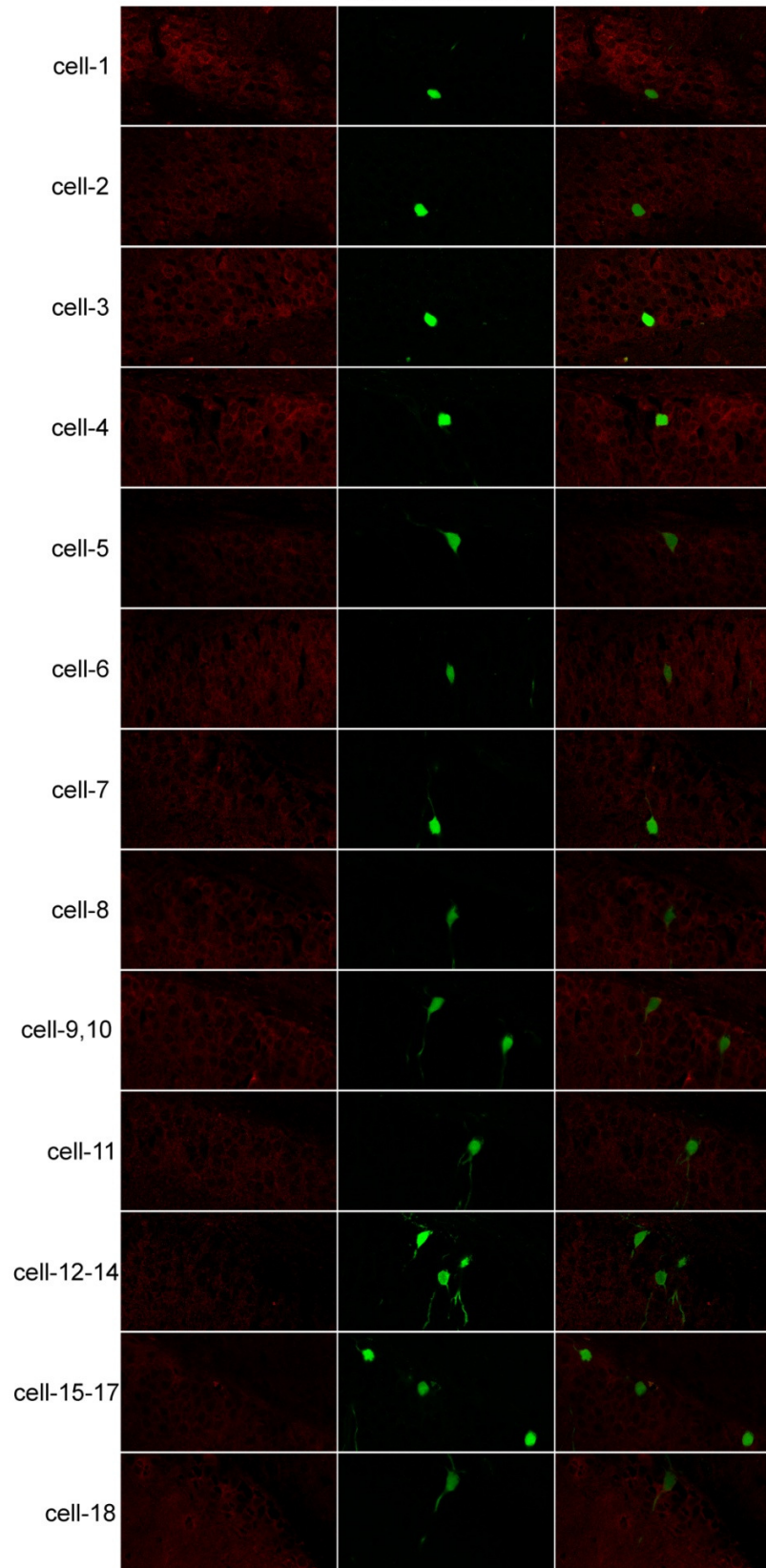

PTZ-untreated SE

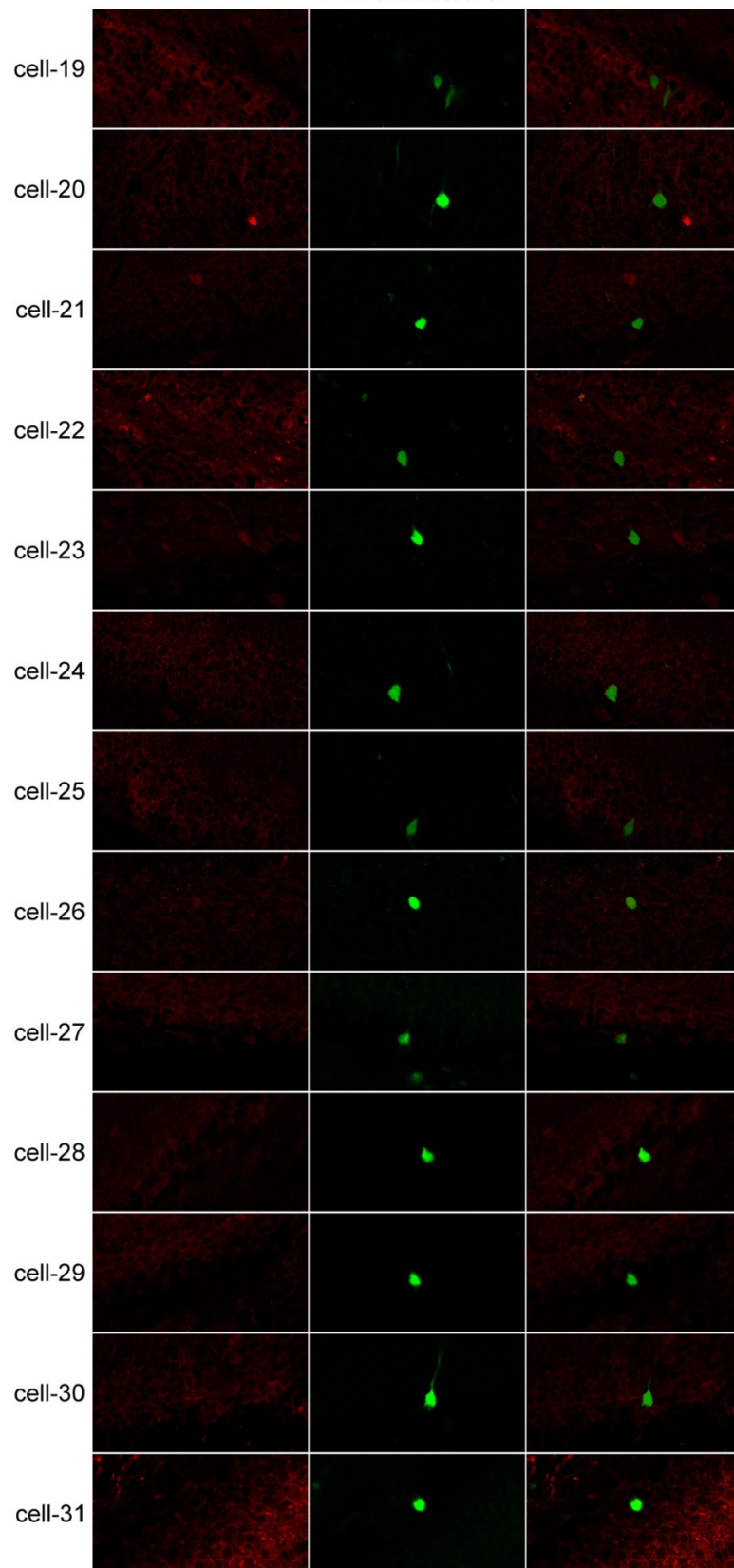

PTZ-untreated SE

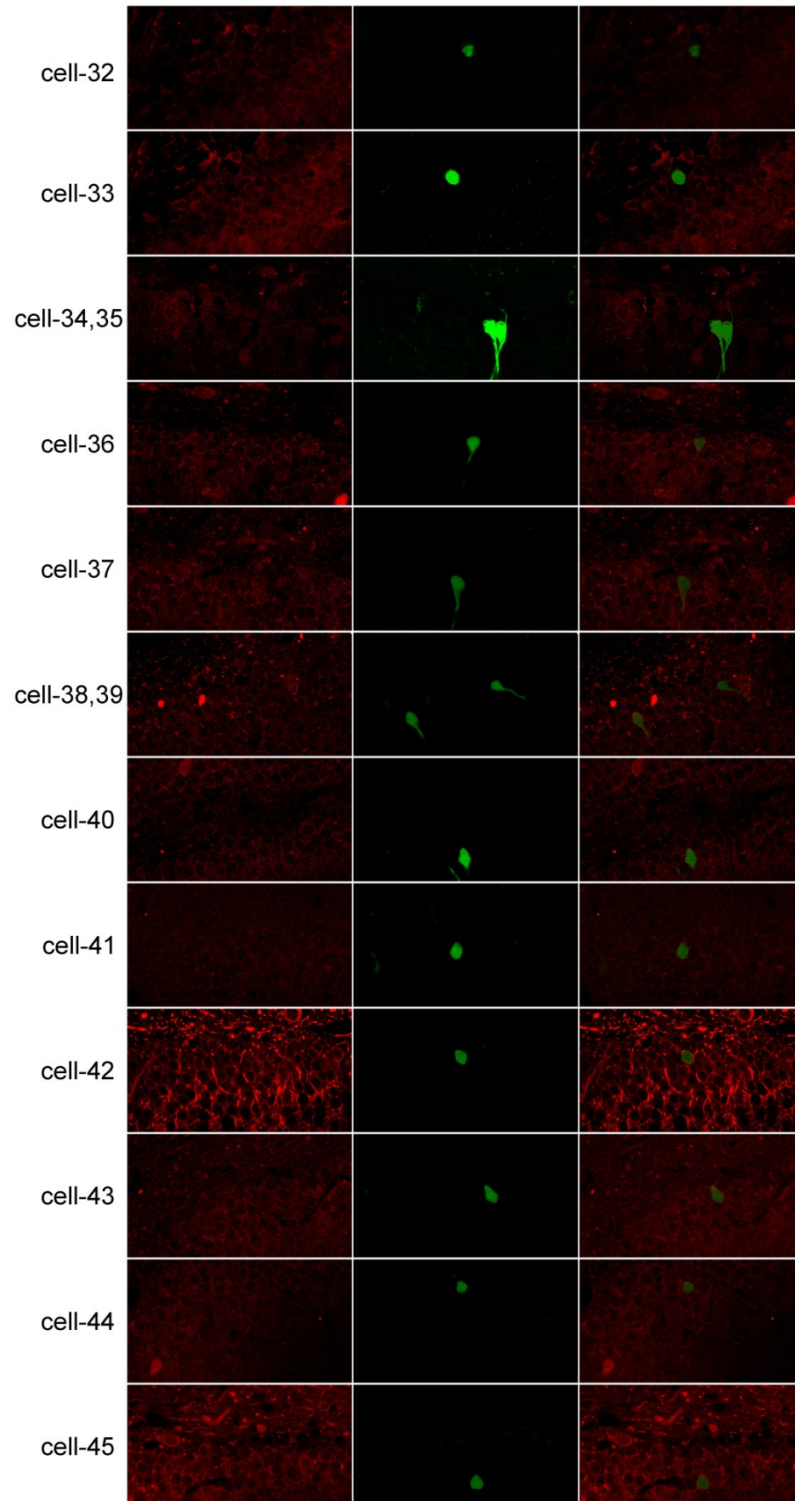

PTZ-untreated SE

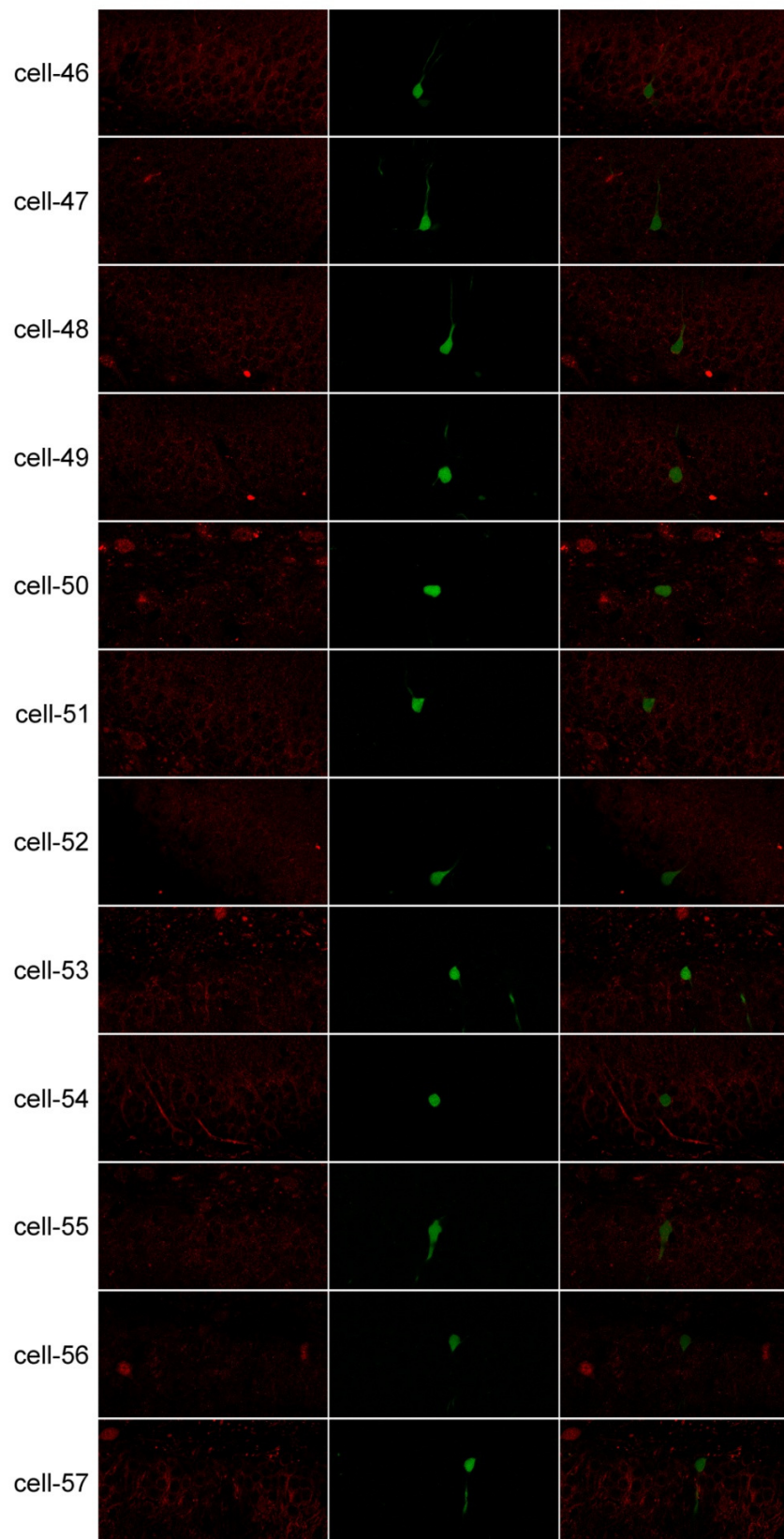

PTZ-untreated SE

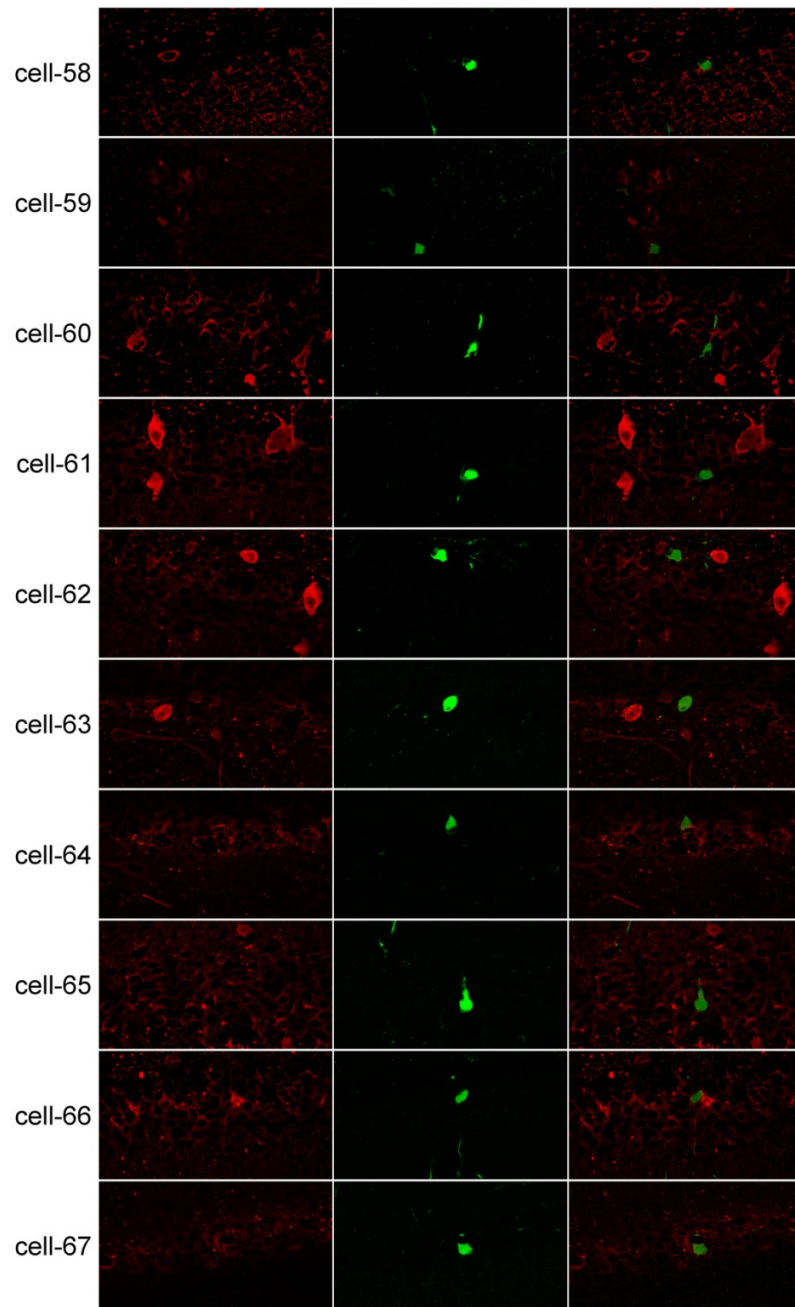

Supplement: Supplementary file 4 [file DataSheet4.PDF]

PTZ-treated SE

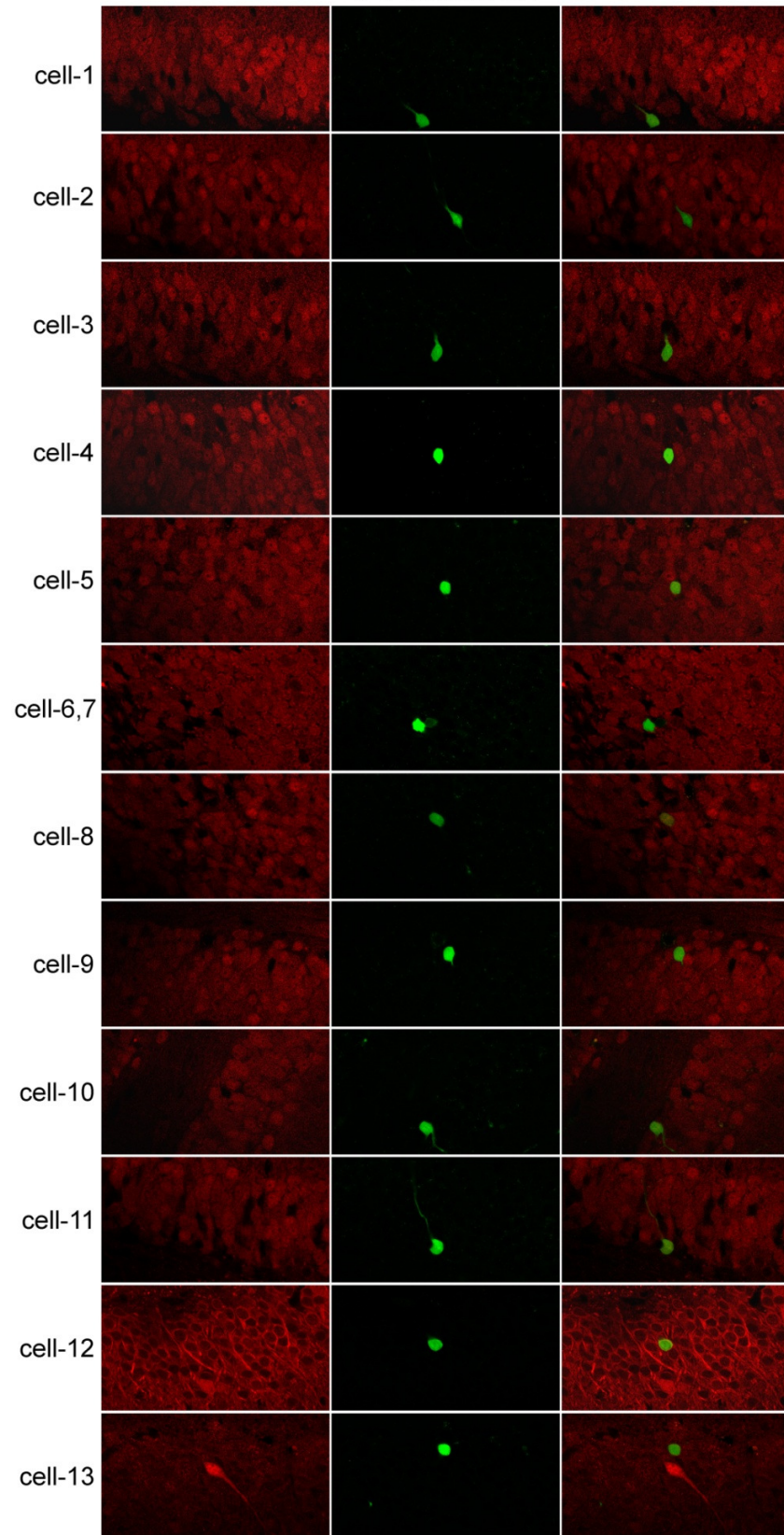

PTZ-treated SE

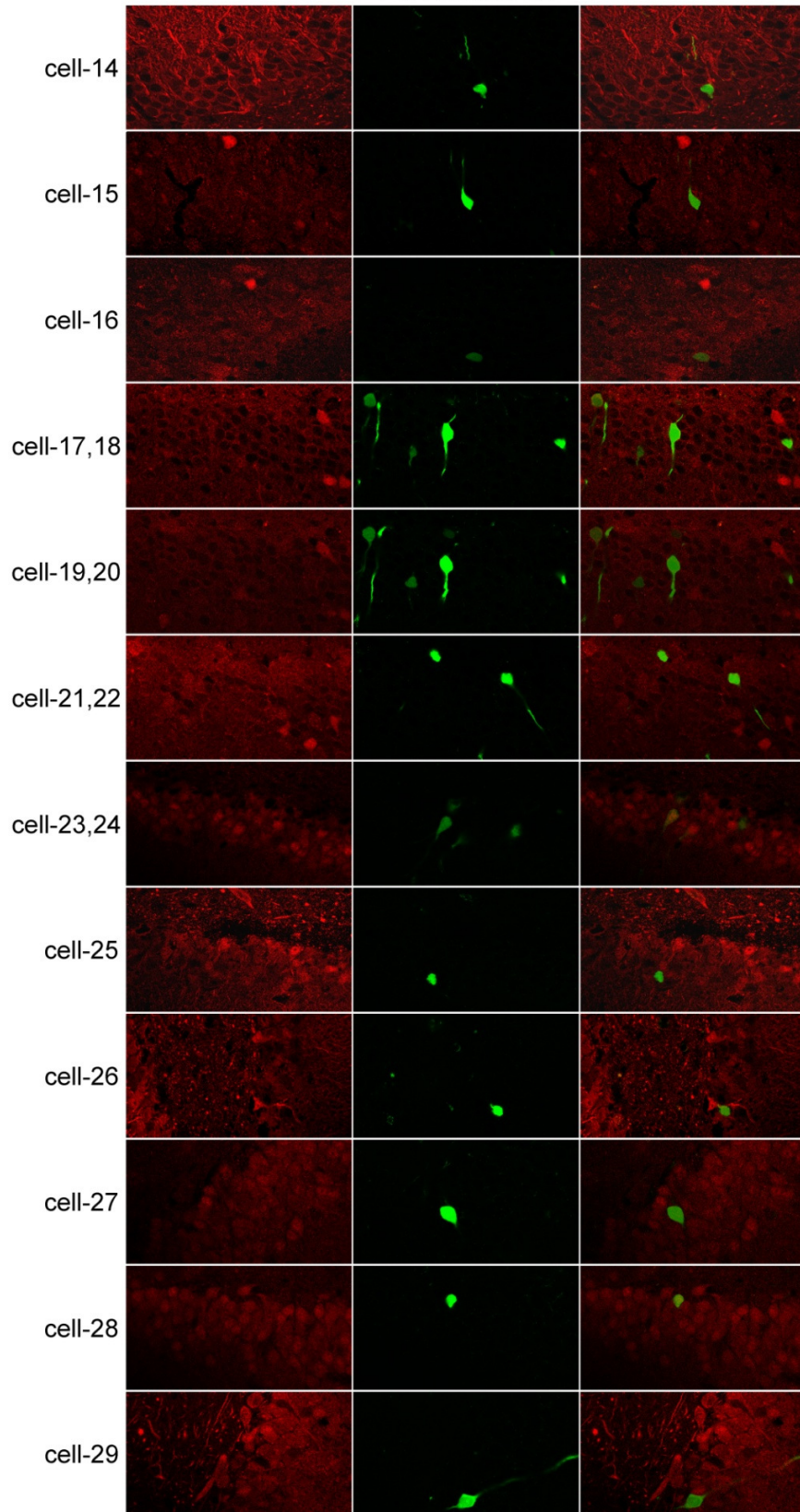

PTZ-treated SE

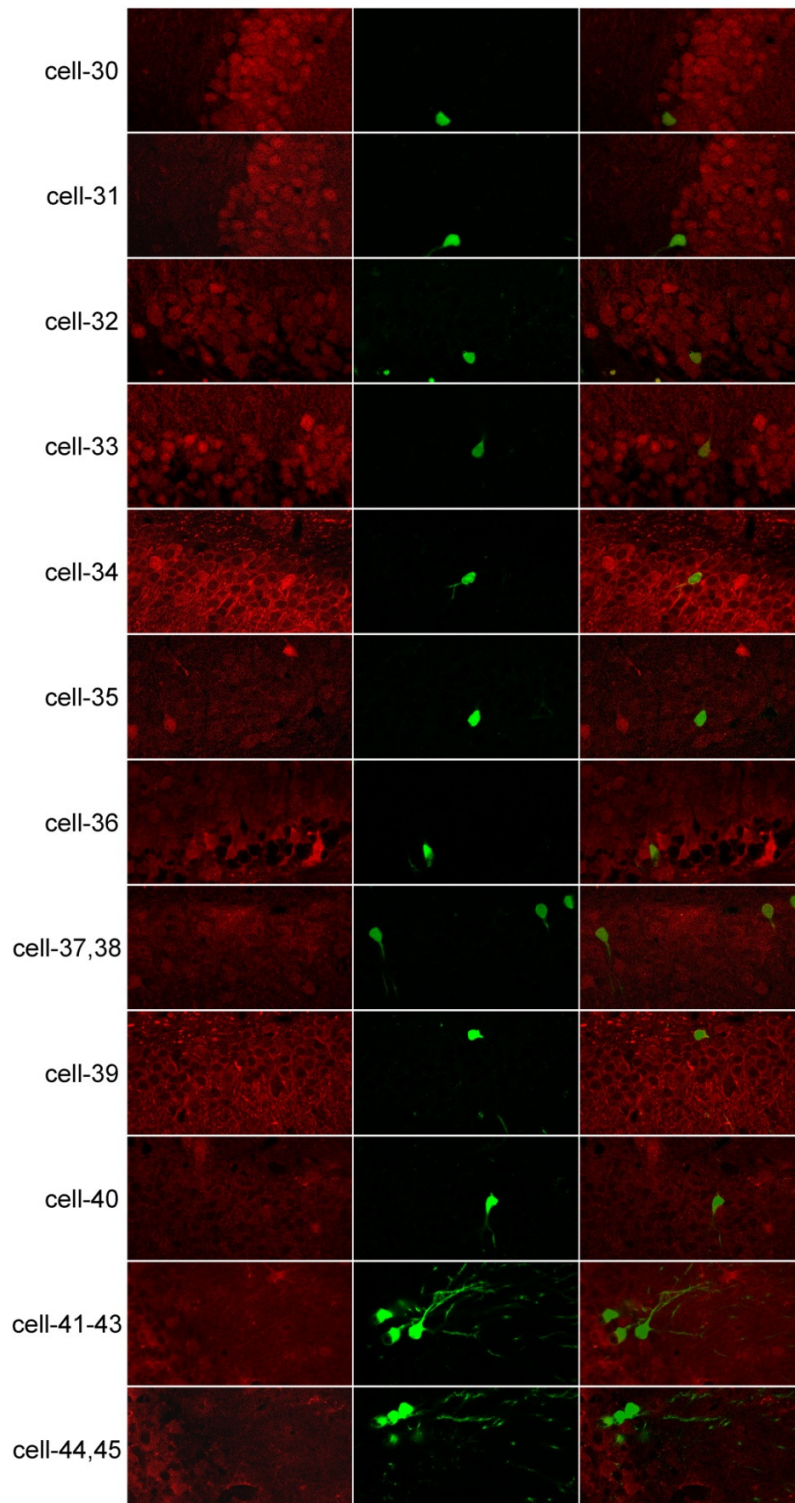

PTZ-treated SE

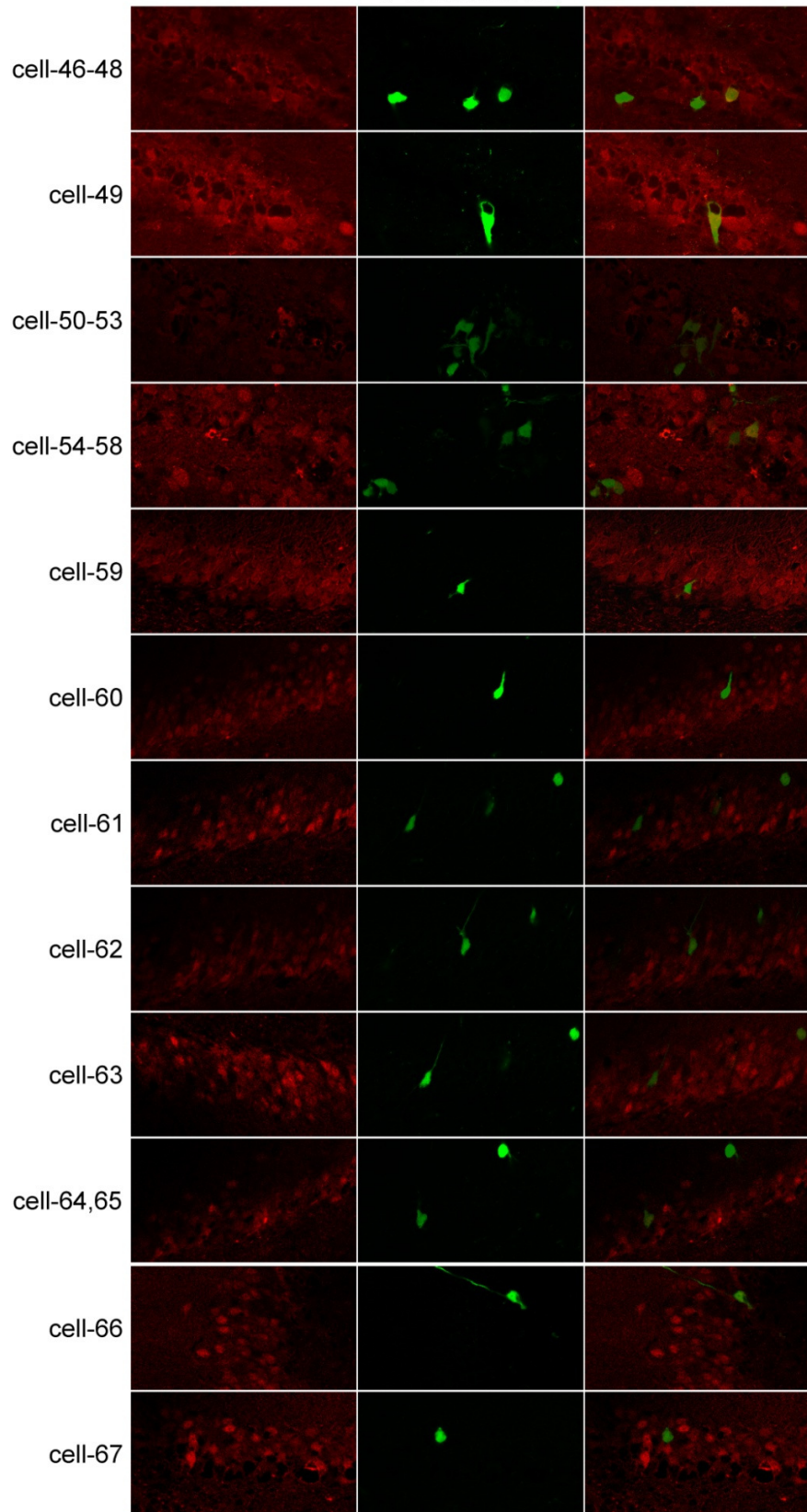

PTZ-treated SE

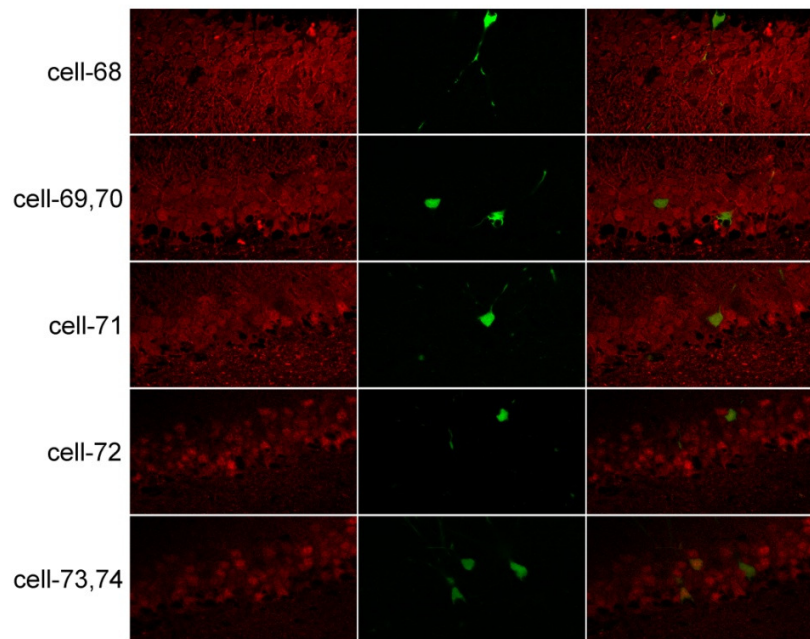

Supplement: Supplementary file 5 [file DataSheet5.PDF]
