# Supplementary material for: Dendritic morphology, synaptic transmission, and activity of mature granule cells born following pilocarpine-induced status epilepticus in the rat
Source: Front Cell Neurosci. 2015 Oct 7;9:384. doi: 10.3389/fncel.2015.00384 (PMC4596052; doi:10.3389/fncel.2015.00384)
Supplement: Supplementary file 3 [file DataSheet3.PDF]

PTZ-treated control

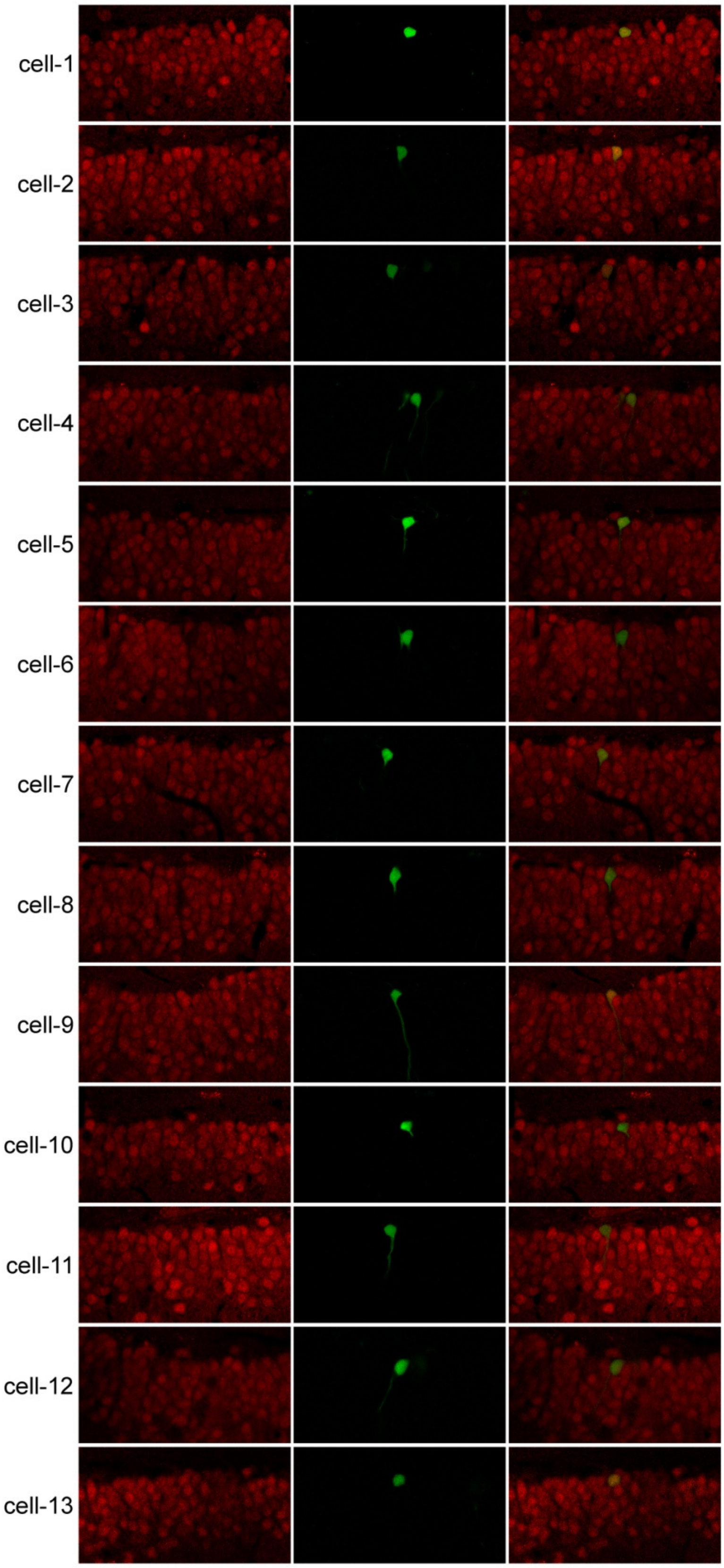

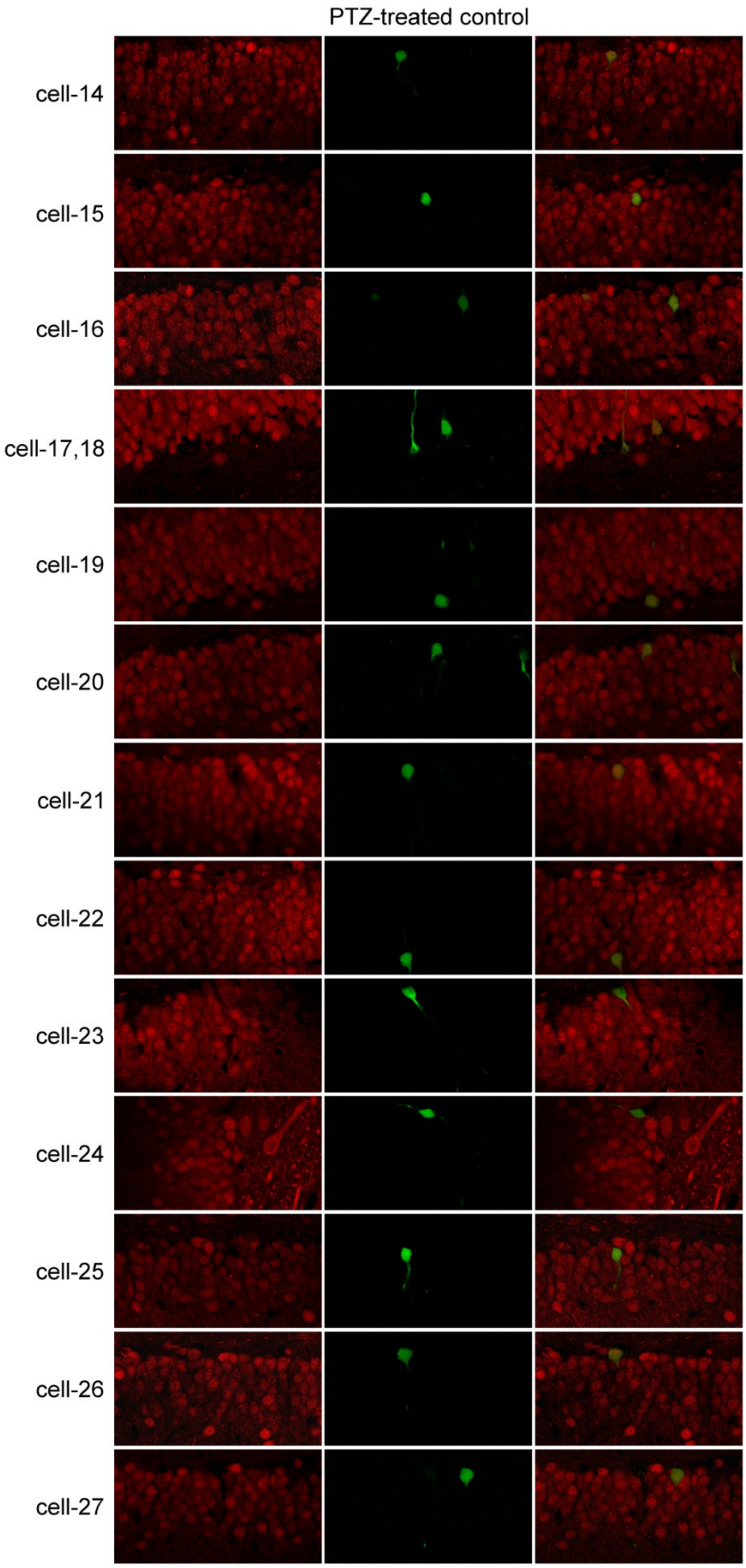
PTZ-treated control
cell-14
cell-15
cell-16
cell-17,18
cell-19
cell-20
cell-21
cell-22
cell-23
cell-24
cell-25
cell-26
cell-27

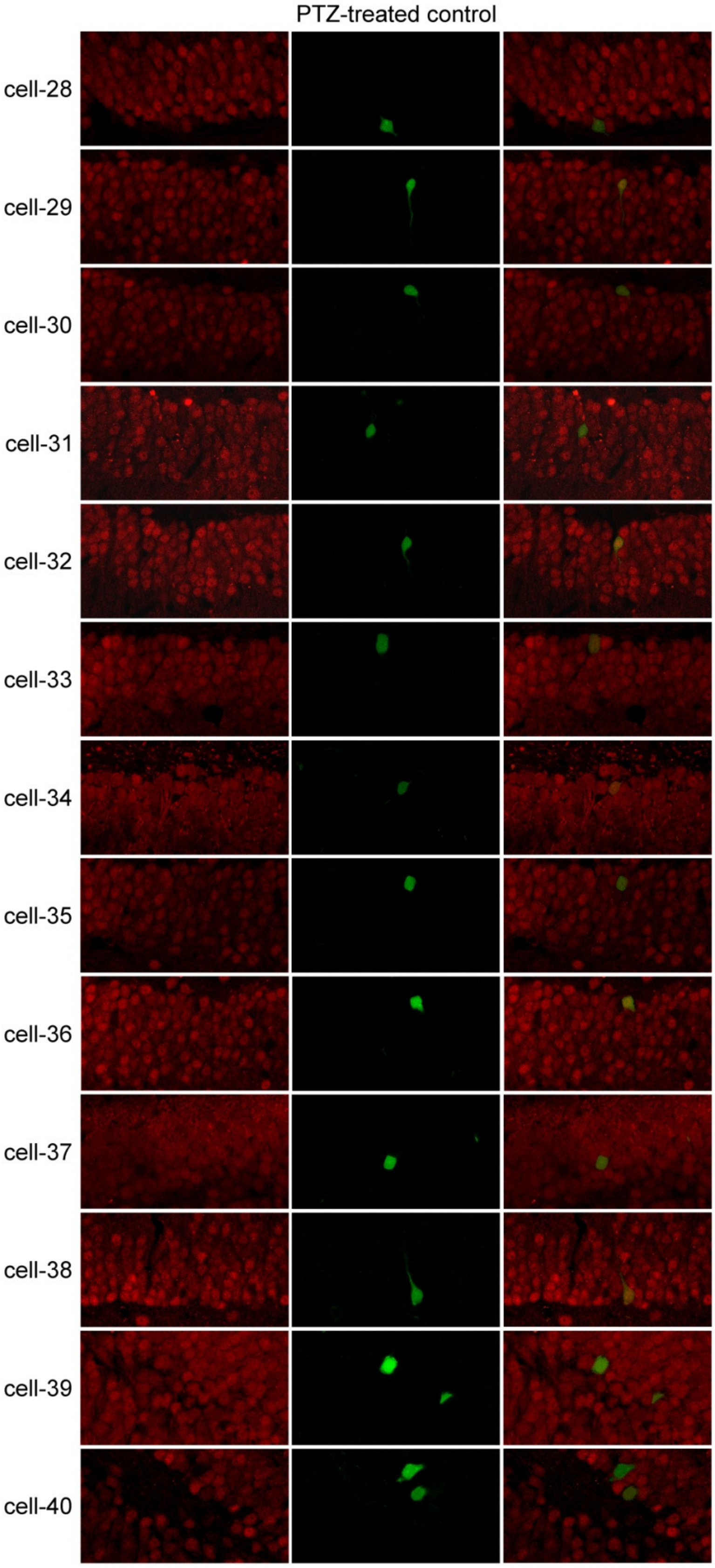
PTZ-treated control
cell-28
cell-29
cell-30
cell-31
cell-32
cell-33
cell-34
cell-35
cell-36
cell-37
cell-38
cell-39
cell-40

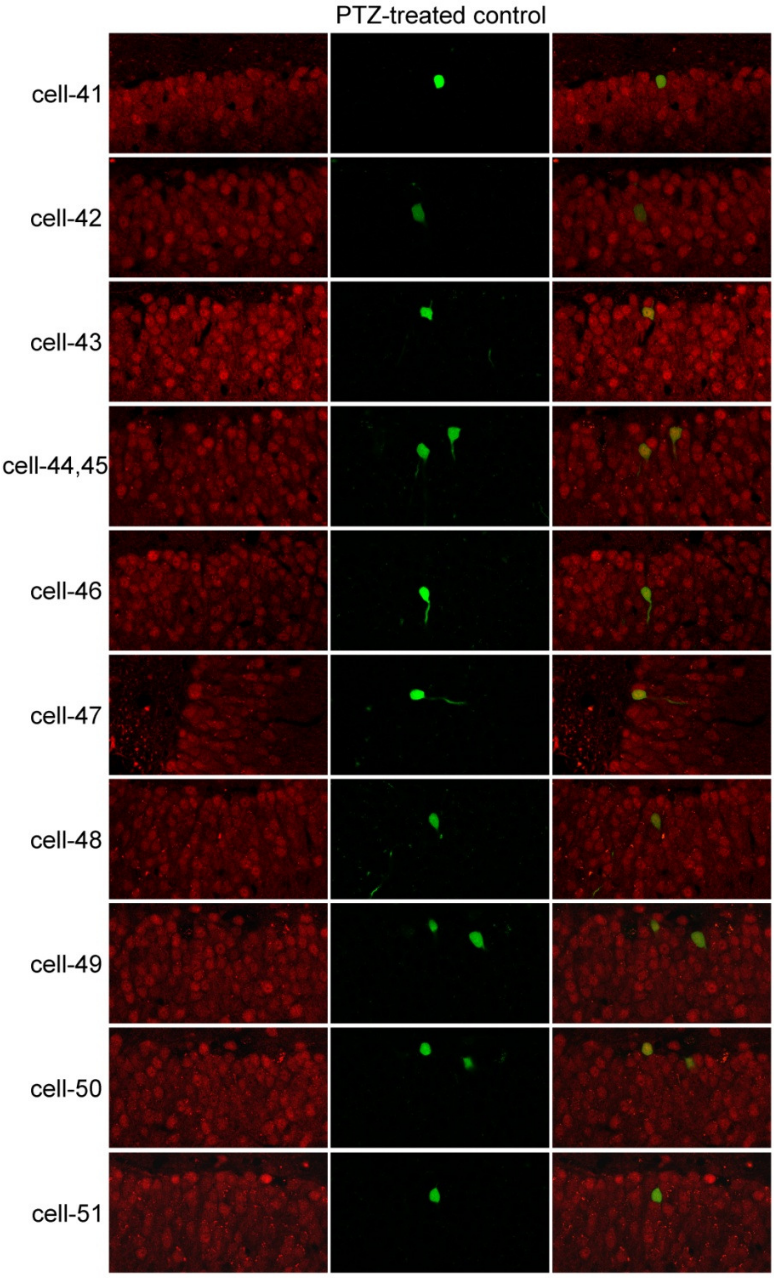
PTZ-treated control
cell-41
cell-42
cell-43
cell-44,45
cell-46
cell-47
cell-48
cell-49
cell-50
cell-51

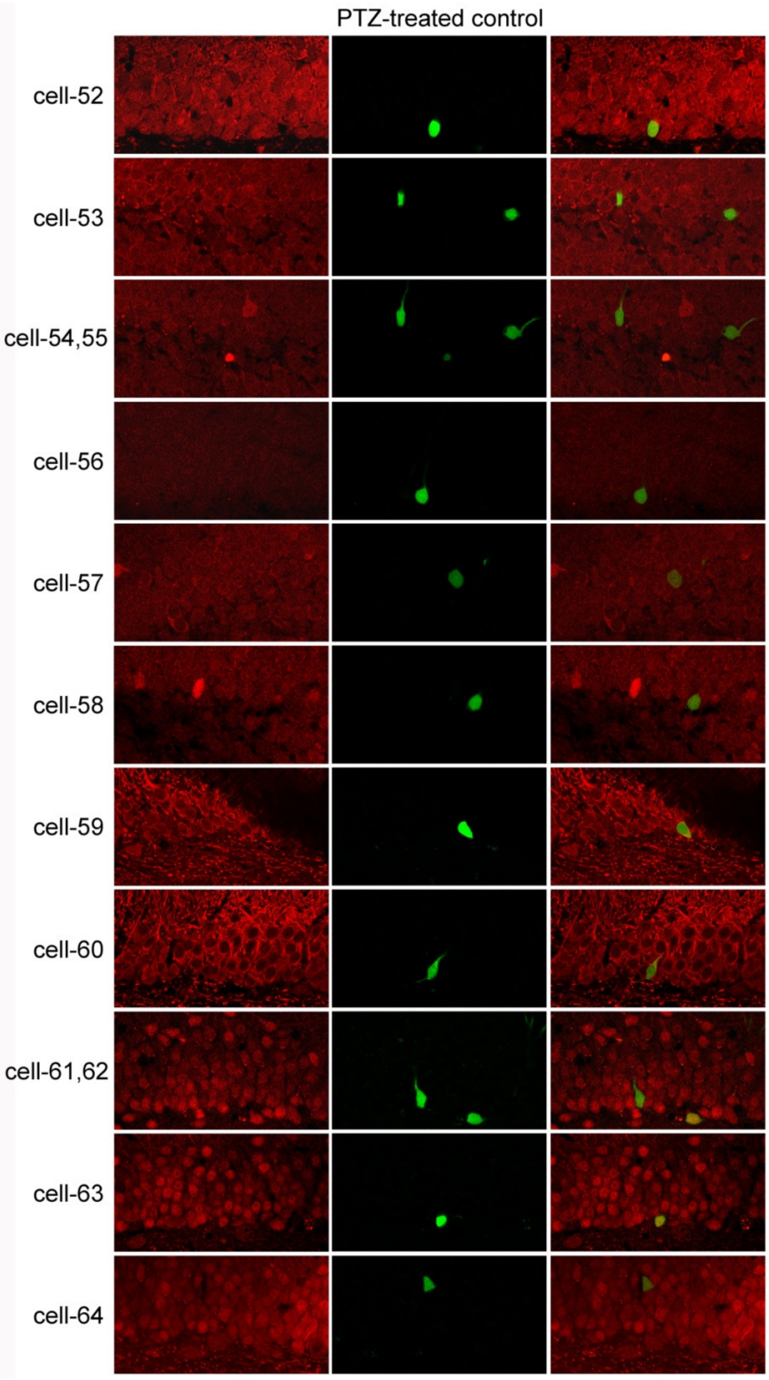
PTZ-treated control
cell-52
cell-53
cell-54,55
cell-56
cell-57
cell-58
cell-59
cell-60
cell-61,62
cell-63
cell-64

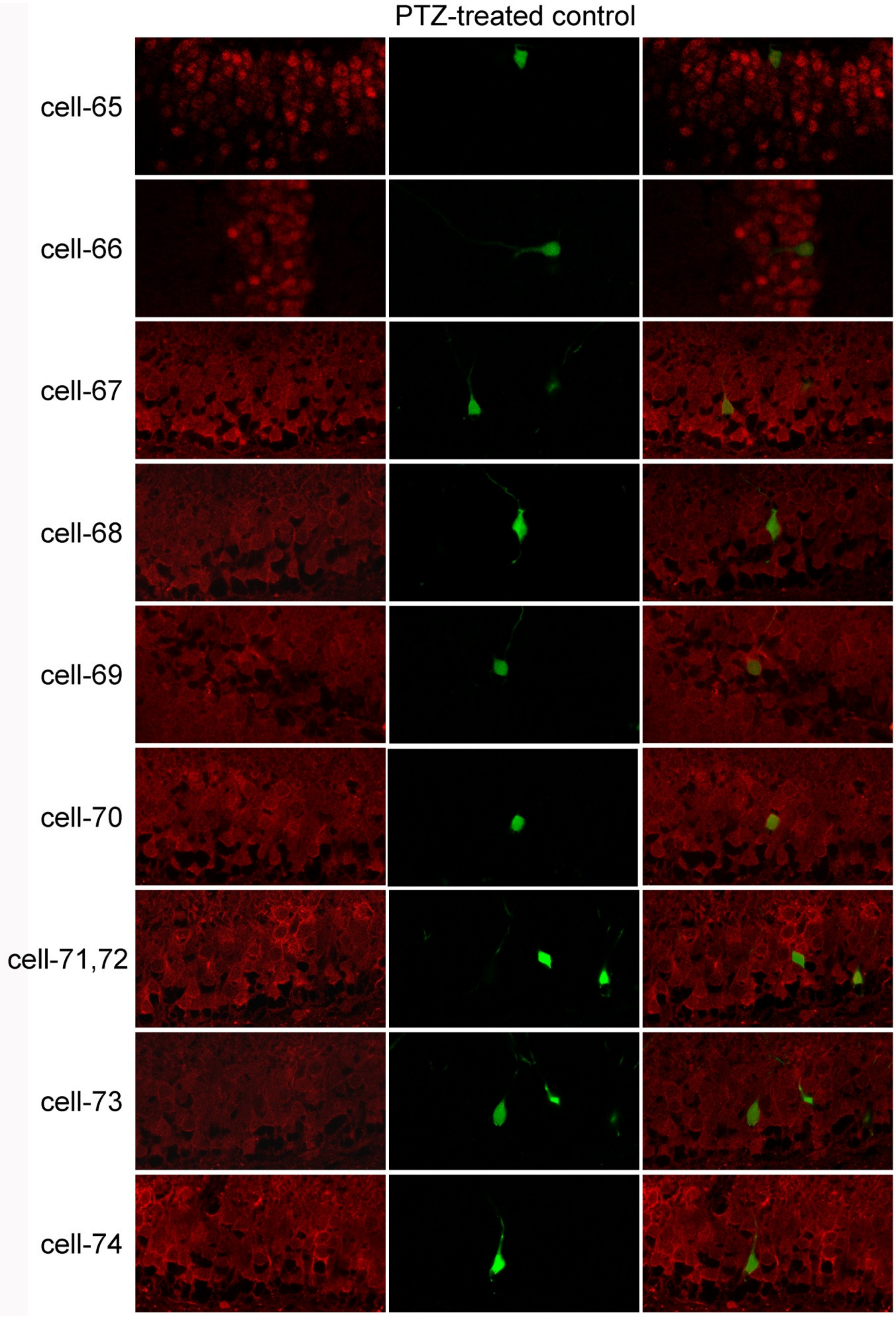
PTZ-treated control
cell-65
cell-66
cell-67
cell-68
cell-69
cell-70
cell-71,72
cell-73
cell-74
